# Monthly Alternations of Core Plant Species in Dynamic Plant‐Pollinator Networks of an Urban Botanical Garden

**DOI:** 10.1002/ece3.71822

**Published:** 2025-07-17

**Authors:** Xiang‐Ping Wang, Shi‐Ran Gu, Zhong‐Tao Zhao, Shi‐Jin Li, Miao‐Miao Shi, Tie‐Yao Tu

**Affiliations:** ^1^ South China Botanical Garden Chinese Academy of Sciences Guangzhou China; ^2^ South China National Botanical Garden Guangzhou China

**Keywords:** core species, dynamic, exotic plants, native plants, plant‐pollinator networks, urban botanical garden

## Abstract

Urban systems, particularly botanical gardens, often comprise a lot of exotic plant species that can integrate into local plant‐pollinator networks, influencing their temporal structural dynamics. However, revealing how plant‐pollinator interactions are continuously reshaped and how the roles of native plant and exotic plant species within networks alter over time remains a significant challenge. Here, we reconstructed monthly plant‐pollinator interaction networks for 12 months within an urban botanical garden. We focused on the monthly variations in the structure of pollination networks and the roles of native and exotic plant species. The results showed that the dynamics of plant‐pollinator interactions are characterized by significant changes in network structure and species alternations, which have substantial impacts on community processes. Each month, the plant‐pollinator network can be divided into several modules of closely interacting plants and pollinators, and these modules form complex fission‐fusion dynamics across the year. Monthly dynamic changes in plant‐pollinator network structure led to alternations of plant species occupying core positions within the networks. The core plant species in the pollination networks alternated between native and exotic species across the 12 months, suggesting that plant species can be core species independently of their origin in urban plant‐pollinator networks. Therefore, the roles of native and exotic plant species in plant‐pollinator networks can only be fully detected and understood from the perspective of time‐scale dynamics. These results suggest that information on the dynamic changes in plant‐pollinator network structure is critical for understanding the temporally varying role of core species in urban ecosystems.

## Graphical abstract

The structure of the plant‐pollinator network in the urban botanical garden is dynamic. The network can be divided into some modules of closely interacting species each month. Dynamic changes in network structure led to alterations of core plant species. The core plant species in networks alternated between native and exotic species. Plant species can be core species independently of their origin in urban networks.

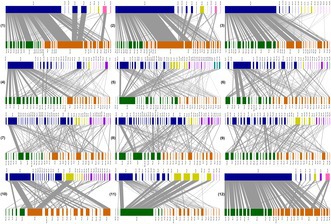

## 1 Introduction

Interactions between plants and pollinators constitute one of the most crucial interspecific interactions within ecosystems. Analyzing the plant‐pollinator networks structure can illuminate specific species or interactions that significantly influence ecosystem stability and function (Toju et al. 2017). Recent research has revealed that plant‐pollinator networks exhibit more pronounced temporal dynamics than previously recognized (Olesen et al. 2008, 2011; Rasmussen et al. 2013; CaraDonna et al. 2017, CaraDonna et al. 2021; Wang et al. 2020, 2021, 2024; Hervías‐Parejo et al. 2023; Magrach et al. 2023). Phenological dynamics of species drive the turnover of plant and pollinator species compositions within a community over time, inevitably altering the structure of plant‐pollinator networks (Poisot et al. 2012; CaraDonna et al. 2017; Wang et al. 2020, 2021). This species turnover influences the formation of interactions between plants and pollinators, ultimately reshaping the structure of the whole community (CaraDonna et al. 2017). Furthermore, even when plant and pollinator compositions remain stable within a community, the interactions between them can still change over time through network rewiring (Poisot et al. 2012). To maintain interactions when both partners are present but not interacting, species can alter their partners over time, potentially modifying their roles within the plant‐pollinator networks (Vázquez et al. 2009; Poisot et al. 2012, 2014; CaraDonna et al. 2017, 2021). However, our understanding of how structures of plant‐pollinator interaction networks themselves change over time remains limited.

In urban ecosystems, especially botanical gardens, the existence of numerous exotic plant species can help maintain urban biodiversity (Quistberg et al. 2016; Baldock et al. 2019; Tew et al. 2021). However, their roles in urban ecosystems have been controversial. Exotic plant species typically exhibit long flowering times (Bjerknes et al. 2007; Pyšek and Richardson 2007), making them an abundant food source for pollinators in urban gardens (Salisbury et al. 2015; Baldock et al. 2019; Tew et al. 2021; Anselmo et al. 2023; Silva et al. 2023). For instance, an exotic tree, *Bombax ceiba* , provided food for 38 bird species in an urban ecosystem (Silva et al. 2023). Exotic plant species frequently interact with highly diverse pollinator species, facilitating their establishment in communities and enabling successful integration into local plant‐pollinator networks, thereby shaping their dynamics (Traveset and Richardson 2014; Thompson and Knight 2018; Arroyo‐Correa et al. 2020; Marquardt et al. 2021; Kovács‐Hostyánszki et al. 2022; Wang et al. 2023). Moreover, exotic plant species can occupy key positions in pollination networks, constituting a core of generalist interactions, interacting with numerous native pollinators, which causes many other species to become more dependent on them (Stouffer et al. 2014; Arroyo‐Correa et al. 2020; Zaninotto et al. 2023). Conversely, other research has demonstrated that native plant species support a substantially larger abundance of native pollinators compared to exotic plants, attributed to their shared phylogenetic history (Pardee and Philpott 2014; Salisbury et al. 2017; Lowenstein et al. 2019; Staab et al. 2020; Cecala and Wilson Rankin 2022; Fenoglio et al. 2023; Nakamura et al. 2023; Prendergast 2023). Native plant species continue to play core positions in pollination networks (Nakamura et al. 2023). Additionally, research has found that generalist pollinators are expected to preferentially visit exotic plants, while specialist pollinator species still rely on native plant species in urban green space (Seitz et al. 2020; Wenzel et al. 2020; Egawa and Koyama 2023). Consequently, the roles of native plant and exotic plant species in plant‐pollinator interaction networks may depend on the species composition in the community at specific times.

Plant species richness and flower availability have been demonstrated to be more crucial than geographic origin for maintaining the abundance and richness of pollinators in urban ecosystems (Salisbury et al. 2015; Wenzel et al. 2020; Berthon et al. 2021). Among all flowering plants in urban botanical gardens, the number of native and exotic plant species that flower simultaneously is undoubtedly dynamic over time. These phenological dynamics of native and exotic plant species throughout the year lead to turnover in plant species compositions within communities, inevitably altering the plant‐pollinator network structure (CaraDonna et al. 2017; Wang et al. 2020, 2023). Accordingly, the core (hub) plant species would also change in temporal networks, imposing effects on diverse pollinator species and potentially determining community constancy. Despite the dynamic nature of plant‐pollinator interactions, our understanding of the roles of native plant and exotic plant species in these networks often derives from interaction data, which are based on observations of interactions between plants and pollinators that occur over a short period or through the entire flowering season (Poisot et al. 2012; Oliver et al. 2019; Kaiser‐Bunbury et al. 2011). Long‐term interaction data cannot capture the dynamics of plant‐pollinator interaction build‐up, and the structure of these networks can differ considerably from that of time‐resolved networks (Rasmussen et al. 2013). Decomposing plant‐pollinator interaction networks into finer time frames can help us infer how communities assemble over time and how species roles change in the networks. Although the plant‐pollinator interactions are widely reported to be temporally dynamic, time series data with species‐level resolution are still rare. Understanding the dynamic roles of native plant and exotic plant species in ecological processes is crucial for reducing biodiversity loss (Traveset and Richardson 2014). However, to our knowledge, although the potential importance of native and exotic plant species interactions in urban ecosystems is acknowledged, the dynamic variation of the roles of native and exotic plant species in plant‐pollinator interaction networks has not been explored.

Comprehensive data on the dynamics of interactions between plants and pollinators and core plants in urban ecosystems will enhance our understanding of community‐level phenomena. This study conducted year‐round observations in a botanical garden that blooms year‐round and contains many exotic plant species, providing an excellent opportunity to explore the temporal dynamics of plant and pollinator interactions and the role of plants in networks over time. The aim was to understand how plant‐pollinator networks containing different native plant and exotic plant species are dynamically reshaped and how the role, that is, species contribution to connectivity within and among modules, of native and exotic plant species changes within plant‐pollinator networks over time. The structure, interaction dissimilarity, and the role of plants within plant‐pollinator interaction networks were investigated across twelve months. The hypothesis tested was that the core species in the plant‐pollinator networks would alternate between native and exotic plant species over time.

## 2 Materials and Methods

### 2.1 Study Site

A field investigation was conducted at the South China Botanical Garden (SCBG) in Guangzhou, China (http://www.scbg.ac.cn). The SCBG covers an area of approximately 282 ha and contains over 17,000 plant species, including trees, shrubs, and herbs. Notably, the plant species in the SCBG have diverse origins, with some being native and others being exotic. The botanical garden exhibits continuous flowering throughout the year. Four fixed open‐air sites with high plant species diversity were chosen for pollination observation within the SCBG. Based on the Catalog of Alien Plants in China dataset (Lin et al. 2022; Hao and Ma 2022), we can accurately classify whether individual plants in the botanical garden are native or exotic species. The dataset is accessible via the following links: https://www.scidb.cn/s/qaUZNb; http://doi.org/10.57760/sciencedb.01711; and https://www.biodiversity‐science.net/fileup/1005‐0094/DATA/2022127.zip.

### 2.2 Pollination Observation

A direct observation of flowering plants was conducted on sunny days in each month. The four sites were observed from 8:30 to 15:30 (periods of peak insect activity) for three days, with the observation sequence of the sites altered each day. To minimize interference from tourists, observations and data collection were performed on weekdays. Interactions between flowers and pollinators were recorded by a single observer slowly walking through each site. We observed flowering plant species in a random order at each site during the observation period. We captured all flower visitors that contacted flowers' stigma and/or anthers using an entomological net and transferred them to centrifuge tubes containing ethyl alcohol in the field. Bird pollinators were not captured due to their rarity and difficulty in catching; instead, their numbers were recorded, and photographs were taken. The time spent at each site, including the time required to process the captured pollinators, varies according to pollinator and flower abundance. Consequently, the number of hours needed to sample all four sites varies depending on flowering phenology and the assemblage of pollinators. The pollinators' abundance within each month was evaluated using the number of individuals recorded at four sites. A combination of morphological and molecular techniques was employed to identify the flower visitors. Common flower visitors, such as *Apis cerana* , *Xylocopa appendiculata* , and most butterfly species, were initially identified to the species level based on their distinct morphological features. Molecular identification was performed to identify species that could not be morphologically confirmed by using mitochondrial cytochrome c oxidase subunit I genes for barcoding. We extracted DNA from one or two legs for each insect specimen. These specimens were then preserved in ethyl alcohol, labeled, and deposited at the laboratory of the SCBG. Sequences were submitted to GenBank and the GBOL reference database. The sequences were then searched in the Barcode of Life Data Systems. These species were assigned to species, genus, or family level when the similarity between the specimen and BOLD records matched 95%–100%, 90%–95%, or lower than 90%, respectively.

### 2.3 Plant and Pollinator Diversity

**FIGURE 1:**
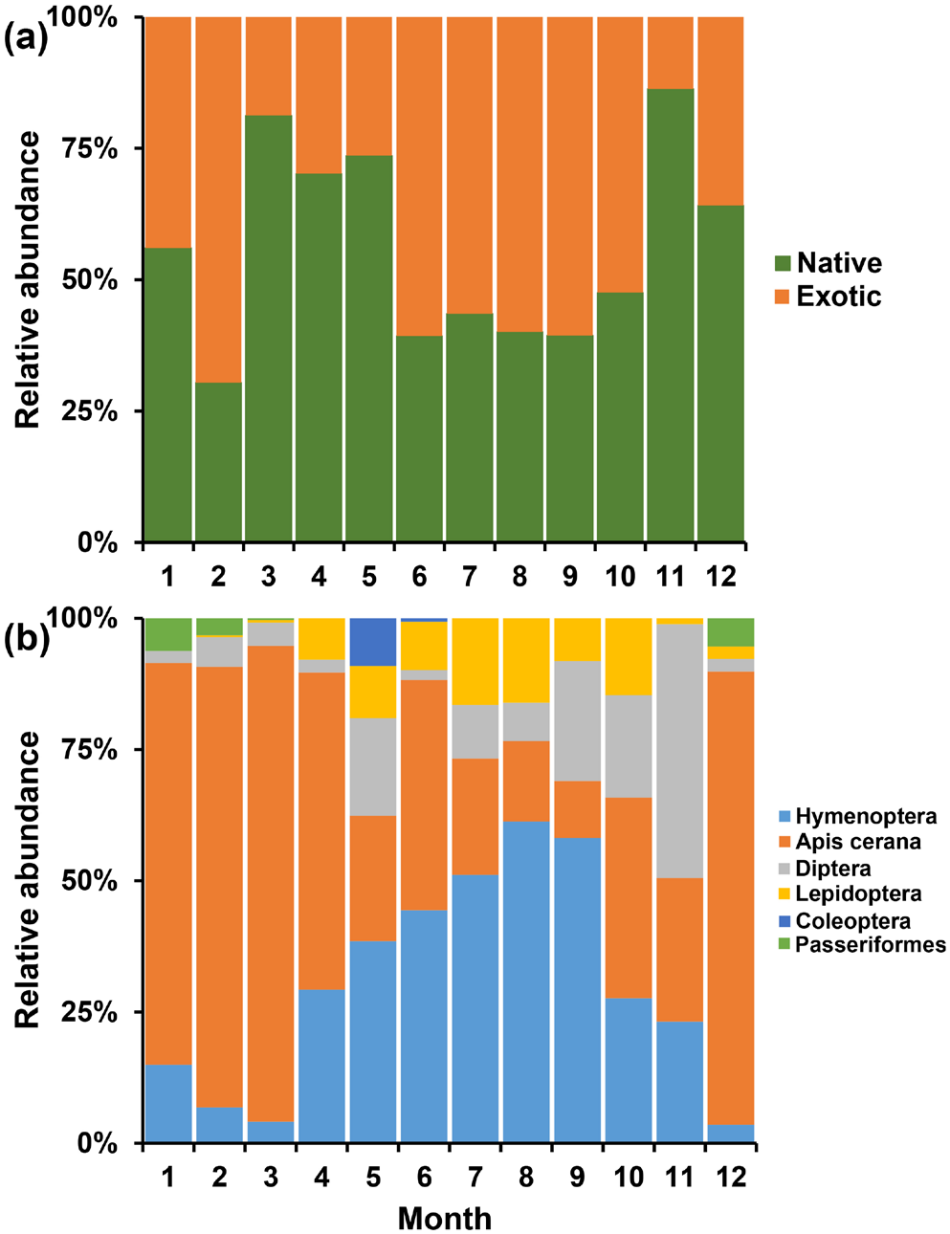
Native and exotic plant species and pollinator species diversity across 12 months in the SCBG. (a) Native and exotic plant species composition. The composition of native and exotic plant species is shown for each month. (b) Pollinator species composition. The composition of pollinator species is shown for each month. See Figure S2 for the number of native and exotic plant species and pollinator species in each month.

Native and exotic plant species compositions were visualized across 12 months using a bar graph (Figure 1). Similarly, the order‐level pollinator compositions were presented in a bar graph according to the number of species. Pollinator species were categorized into five orders: Hymenoptera, Diptera, Lepidoptera, Coleoptera, and Passeriformes. To investigate the variation in pollinator compositions (at both species and order levels) across native and exotic plant species and months, we performed a permutational multivariate analysis of variance using the vegan package in R 4.1.3 with 1000 permutations (Anderson 2001; Oksanen et al. 2007). The order‐level composition of pollinators was set as the response variable, and months, plant species, and plant origin (native or exotic) were set as explanatory variables.

### 2.4 Plant‐Pollinator Interaction Network Structure

Twelve quantitative plant‐pollinator networks were constructed at the species level, one for each month. The interaction strength between plants and pollinators in each network was defined as the number of pollinators visiting each plant species. The plant‐pollinator interaction networks for each month were visualized using the bipartite package (Dormann et al. 2021) in R 4.1.3. To evaluate the monthly variation in the structure of the plant‐pollinator networks, we calculated four network‐level indices for each of the 12 networks. The weighted connectance (WC) was used to calculate network connectance. The H2′ was employed to measure community‐level interaction specialization. The weighted NODF was used to calculate network nestedness, which represents the degree to which specialist species interact with subsets of the partners of generalist species. Barber's modularity index of network compartmentalization was used to calculate network modularity (Barber 2007). The indices of connectance, H2′, and weighted NODF were calculated using the bipartite package in R 4.1.3. For modularity, we first compared three algorithms (‘Louvain’, ‘fast greedy’, and ‘Infomap’) for identifying modules (Clauset et al. 2004; Blondel et al. 2008; Rosvall and Bergstrom 2008) using the igraph package (Csardi and Nepusz 2006) in R 4.1.3. The comparison revealed that Louvain and fast greedy algorithms produced the same values. Moreover, the Louvain algorithm yielded higher modularity scores than the fast greedy algorithm, indicating slightly better performance and more optimized inferred memberships (Figure S1). Consequently, the network modularity was calculated using the Louvain algorithm as follows:

$${\text{Barber}}^{\prime }s\;\text{modularity}=\sum_{j=1}^{N_{M}}\left(\frac{m_{j}}{m}-\frac{s_{j}p_{j}}{m^{2}}\right)$$

where *N*_*M*_ represents the modules number detected within the network; *m* denotes the entire links number within the network; *m*_*j*_ signifies links number within the module *j*; *s*_*j*_ represents the sum of the degrees of all plant species in the module *j*; *p*_*j*_ represents the sum of the degrees of all pollinators within the module *j*.

To calculate the degree to which the real network patterns deviated from those randomly generated networks, the values of H2′, weighted NODF, and Barber's modularity were converted into standardized scores as follows:

$$\text{Standardized index}=\frac{X_{\text{observed}}-\text{mean}\;\left(X_{\text{randomized}}\right)}{\mathrm{SD}\;\left(X_{\text{randomized}}\right)}$$

where *X*_observed_ is the detected value of the network index; mean (*X*_randomized_) and SD (*X*_randomized_) are the mean and the standard deviation of the index values of randomized networks. Network connectance was not standardized because it represented the proportion of detected plant‐pollinator interactions to all possible interactions. First, the plant species were shuffled in each random network (1000 permutations) to generate sample‐level randomized networks. Second, these networks were converted into species‐level networks for calculating the network index. Based on the randomization, the Benjamini‐Hochberg adjustment of *p* values (false discovery rate, FDR < 0.05) was used to examine the significance of the standardized H2′, weighted NODF, and modularity.

### 2.5 Interaction Turnover Temporal Variation

To assess the dissimilarity of plant‐pollinator interaction networks over 12 months, the turnover of interaction composition across months was calculated. Interaction turnover defines the degree to which plant‐pollinator interactions are increased or lost over time (Poisot et al. 2012; Legendre 2014; Noreika et al. 2019; Fründ 2021). To elucidate the potential drivers and patterns of temporal turnover in interactions between plants and pollinators, we used Whittaker's beta diversity index to calculate the interaction turnover as follows:

$$\beta_{\mathrm{WN}}=\frac{a+b+c}{\left(2a+b+c\right)/2}-1$$

where a represents the number shared between the two compared networks; b and c denote the links number exist in only one network. The Ruzicka dissimilarity index was used to calculate the interaction turnover between two compared networks using the bipartite package in R 4.1.3. The value of *β*_WN_ ranges from 0 to 1, and a higher value represents a greater turnover. To identify the drivers of differences in interaction turnover between networks, *β*_WN_ was partitioned into two subcomponents: *β*_st_ and *β*_os_. *β*_st_ represented changes in species composition. *β*_os_ represented interaction rewiring, which refers to shared plant/pollinator species interacting with different pollinator/plant species within the compared networks. Interaction rewiring is considered a dissimilarity in species interactions between species shared by both networks, reflecting changes in pollinator partner choice. Furthermore, the interaction turnover caused by variation in species composition (*β*_*s*_) was decomposed into variation in pollinators composition (*β*_ST.h_), plants composition (*β*_ST.l_), and both (*β*_ST.hl_).

### 2.6 Genealogy Analysis of Plant‐Pollinator Network Modules

Closely interacting plant and pollinator species were assembled within network modules based on the analysis using the Louvain algorithm (Clauset et al. 2004). The ‘genealogy’ of these network modules was then estimated across 12 months using the alluvial diagram approach, which was visualized by using the ggalluvial package (Brunson 2020) in R 4.1.3. The resulting graph illustrates the flow and dynamics of plant and pollinator species within these modules over 12 consecutive months, providing insights into the temporal changes in the interactions between these species.

### 2.7 Plant‐Pollinator Interaction Generality and Network Centrality

To examine whether core plant species (native or exotic) changed throughout the months, two species‐level metrics were employed to identify native plant and exotic plant species at central positions in plant‐pollinator networks. The first metric was the d' index of standardized Kullback–Leibler distance of specificity to partners (Blüthgen et al. 2006). The value of d' ranges from 0 to 1, with a higher value indicating a higher specificity of a species. Consequently, the value of 1−*d*′ can be utilized to evaluate the degree to which plant species interact with a wide range of pollinators, denoting interaction generality. This index facilitates the identification of plant species that have excessive impacts on diverse pollinators relative to their flower abundance. We used the bipartite package in R 4.1.3 to calculate the d' index. The second index was betweenness centrality. It measures the degree to which a specified vertex is positioned in the shortest paths linking pairs of other vertices within networks. Betweenness centrality can be employed to assess the degree to which the species is located at the central position for each plant species in the network. Species that are located at the core positions in networks can affect the population dynamics of other coexisting species in communities (Toju et al. 2017). Values of betweenness centrality can be standardized as follows:

$$\text{Standardized betweenness centrality}=\frac{2B}{\left(n-1\right)\left(n-2\right)}$$

where *B* represents the raw value and n denotes the total number in the network. The value of standardized betweenness centrality ranges from 0 to 1, and a value of 0 indicates species positioned at the network's periphery, while a value of 1 signifies species located on the shortest path connecting all vertex pairs (Dormann et al. 2021). Consequently, species exhibiting betweenness centrality values exceeding 0.25 are considered potential mediators for more than a quarter of the vertex pairs. The betweenness centrality index is designed to identify topologically central species in networks, regardless of their abundance. We calculated the betweenness centrality index using the igraph package (Csardi and Nepusz 2006) in R 4.1.3. Subsequently, we assessed the positions of native and exotic plant species on a bidimensional surface where the interaction generality was defined as abscissa and the betweenness centrality as ordinate.

### 2.8 Meta‐Network Analysis

To comprehend the structure of the annual plant‐pollinator network, referred to as a meta‐network, we integrated twelve plant‐pollinator subnetworks representing each month from January to December. In total, the meta‐network comprised 151 plant species and 142 pollinator species. To evaluate the topological roles of native and exotic plant species within and across the modules, we identified modules in the meta‐network using the aforementioned Louvain algorithm. The index of within‐module degree denotes the number of links to other vertices in the same module. The within‐module degree of each plant species was calculated and standardized by using the following formula:

$$z-\text{standardized within}-\text{module degree}\;\left(i\right)=\frac{k_{\text{is}}-{\bar{k}}_{s}}{{\mathrm{SD}}_{\mathrm{ks}}}$$

where *k*_is_ represents the number of links from vertex *i* to other species in the module; $\bar{k}$_*s*_ and SD_ks_ represent the mean and the standard deviation of the within‐module degrees of all vertices in the module.

The index of among‐module connectivity denotes the position of a vertex in the module and in relation to other modules, which can assess the degree to which each species interconnects modules. The among‐module connectivity of each plant species in the meta‐network was calculated by using the following formula:

$$\text{Among}-\text{module connectivity}\;\left(i\right)=1-\sum_{j=1}^{N_{M}}\left(\frac{k_{\mathrm{ij}}}{k_{i}}\right)$$

where *N*_*M*_ represents the number in the network, *k*_*i*_ represents the degree of vertex *i*, and *k*_*ij*_ represents the number of links from vertex *i* to other vertices in module *j*. The values of among‐module connectivity range from 0 to 1, with a value of 0 indicating all links are in the module to which the target vertex belongs, and a value of 1 indicating all links are evenly distributed among all modules. In ecological networks, if a species has a within‐module degree more than 2.5 and an among‐module connectivity more than 0.62, then this species is designated as a ‘super generalist’ (Olesen et al. 2007).

## 3 Results

### 3.1 Plant and Pollinator Diversity

We observed 151 plant species (77 native and 74 exotic plant species) representing 56 families and 142 pollinator species (68 Hymenoptera species, 24 Diptera species, 45 Lepidoptera species, 3 Coleoptera species, and 2 Passeriformes species) in the SCBG. Among the 142 pollinator species, 36% were identified morphologically and 64% molecularly (Table S2). The species compositions of native and exotic plants and pollinators changed dramatically across months (Table 1 and Figure 1). Exotic plants mainly appeared in February and from June to October. The pollinator species compositions shifted considerably throughout the months (Figure 1 and Table 1). The pollinator compositions across the entire year were characterized by a high proportion of Hymenoptera pollinators, with *Apis cerana* being the most abundant pollinator, especially in winter and early spring. Diptera was one of the major pollinators from September to November. From April to October, the proportion of Lepidoptera increased. Coleoptera was observed in May, and two Passeriformes species were observed in December, January, and February.

**TABLE 1:** A PERMANOVA of species‐level and order‐level pollinator compositions was performed by setting months, plant species, and plant origin (native or exotic) as explanatory variables.

Explanatory	df	*F*_model_	*R* ^2^	*p*
Species‐level				
Month	11	2.365	0.053	**0.001**
Plant species	150	1.477	0.453	**0.001**
Plant origin	1	0.554	0.001	0.815
Order‐level				
Month	11	2.338	0.049	**0.001**
Plant species	150	1.781	0.506	**0.001**
Plant origin	1	1.027	0.002	0.370

*Note:* Significant *p*‐value ( < 0.050) are indicated in bold.

### 3.2 Temporal Variation in Plant‐Pollinator Interactions

Species composition dissimilarity between months was primarily attributed to the variation in plant species composition from January to June, and to changes in both plant and pollinator species from August to November (Table 2). Interaction turnover was high between months (mean *β*_WN_ 0.82 ± 0.04 SE), and was predominantly driven by species turnover (mean *β*_ST_ 0.58 ± 0.05) rather than interaction rewiring (mean *β*_OS_ 0.23 ± 0.02). The dissimilarity in species composition among months (mean *β*_S_ 0.57 ± 0.05) was largely influenced by variations in plant species composition (mean *β*_ST.l_ 0.34 ± 0.04) as opposed to changes in pollinator species composition (mean *β*_ST.h_ 0.10 ± 0.03) and changes in both plant and pollinator species (mean *β*_ST.lh_ 0.14 ± 0.03).

**TABLE 2:** Beta diversity of plant‐pollinator interactions across 12 months.

Month	*β*_WN_	*β*_OS_	*β*_ST_	*β*_S_	*β*_ST.l_	*β*_ST.h_	*β*_ST.lh_
January–February	0.54	0.28	0.27	0.28	0.22	0.02	0.02
February–March	0.57	0.32	0.25	0.32	0.21	0.03	0.02
March–April	0.90	0.10	0.79	0.62	0.67	0.03	0.09
April–May	0.91	0.14	0.77	0.71	0.51	0.04	0.22
May–June	0.85	0.18	0.67	0.62	0.42	0.07	0.18
June–July	0.83	0.28	0.55	0.57	0.18	0.26	0.10
July–August	0.78	0.28	0.50	0.49	0.24	0.21	0.05
August–September	0.84	0.29	0.55	0.60	0.22	0.20	0.13
September–October	0.96	0.25	0.72	0.71	0.31	0.14	0.27
October–November	0.93	0.22	0.71	0.74	0.30	0.17	0.24
November–December	0.86	0.18	0.68	0.75	0.37	0.03	0.29
December–January	0.81	0.29	0.52	0.47	0.48	0.03	0.01

*Note:* *β*_WN_, Interaction network dissimilarity; *β*_OS_, Dissimilarity in interaction rewiring; *β*_ST_, Dissimilarity in species composition; *β*_S_, Species composition dissimilarity; *β*_ST.l_, Species composition into changes in plant species composition; *β*_ST.h_, Species composition into changes in pollinator species composition; *β*_ST.lh_, Species composition into changes in both plant and pollinator species.

### 3.3 Plant‐Pollinator Network Dynamics

**FIGURE 2:**
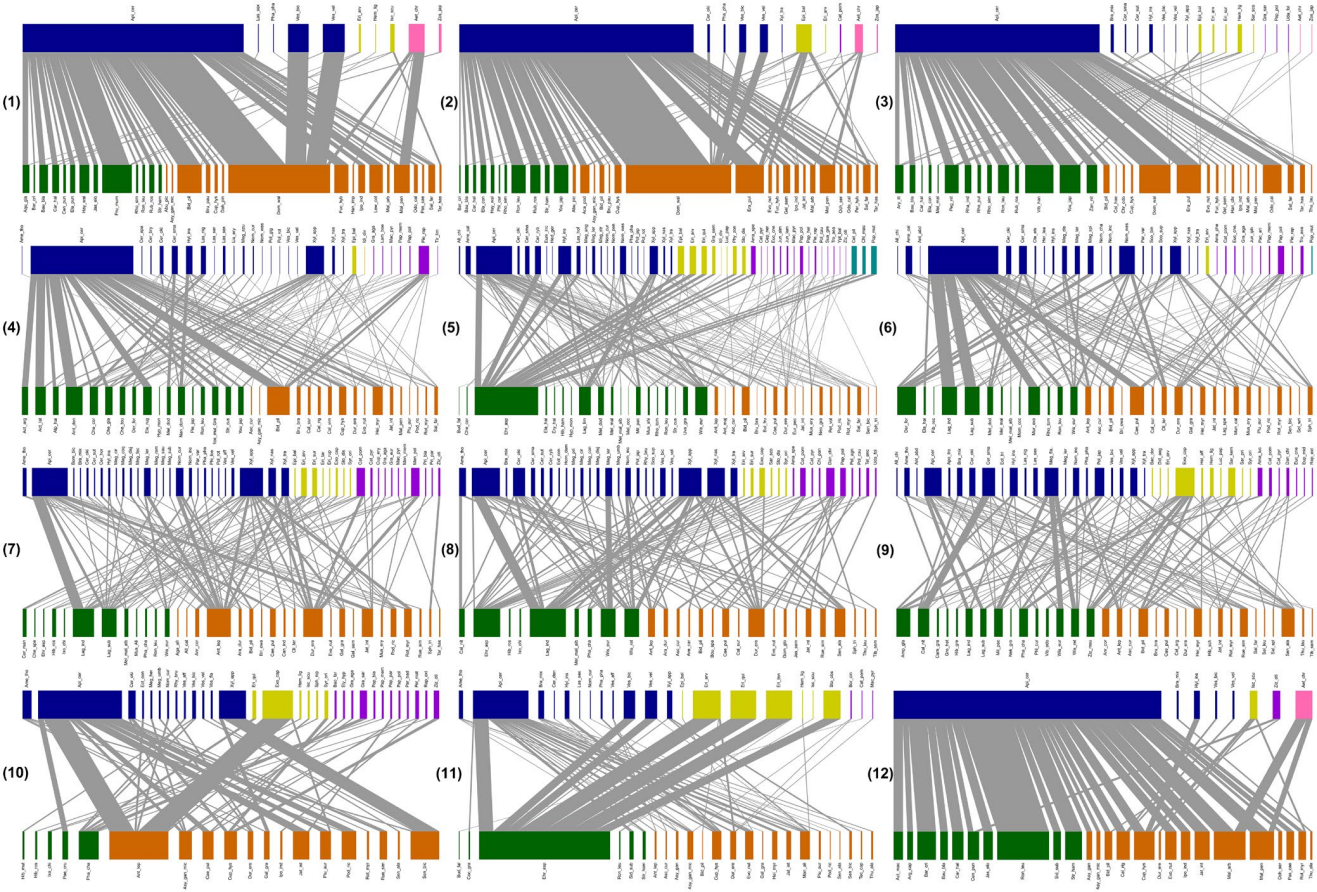
Bipartite networks depicting plant‐pollinator interactions over 12 months at the SCBG. Subplots (1) through (12) represent January to December, respectively. Within each monthly network, the rectangles in the top row symbolize pollinator species, while those in the bottom row represent plant species. The lines connecting the rectangles indicate interactions between plant and pollinator species, with line thickness proportional to the observed number of visits. Plant species are color‐coded as follows: Dark green for native species and dark orange for exotic species. Pollinator species are color‐coded based on their taxonomic order: Dark blue for Hymenoptera, dark yellow for Diptera, dark violet for Lepidoptera, dark cyan for Coleoptera, and pink for Passeriformes. The full names of plant and pollinator species are provided in Tables S1 and S2, respectively.

**FIGURE 3:**
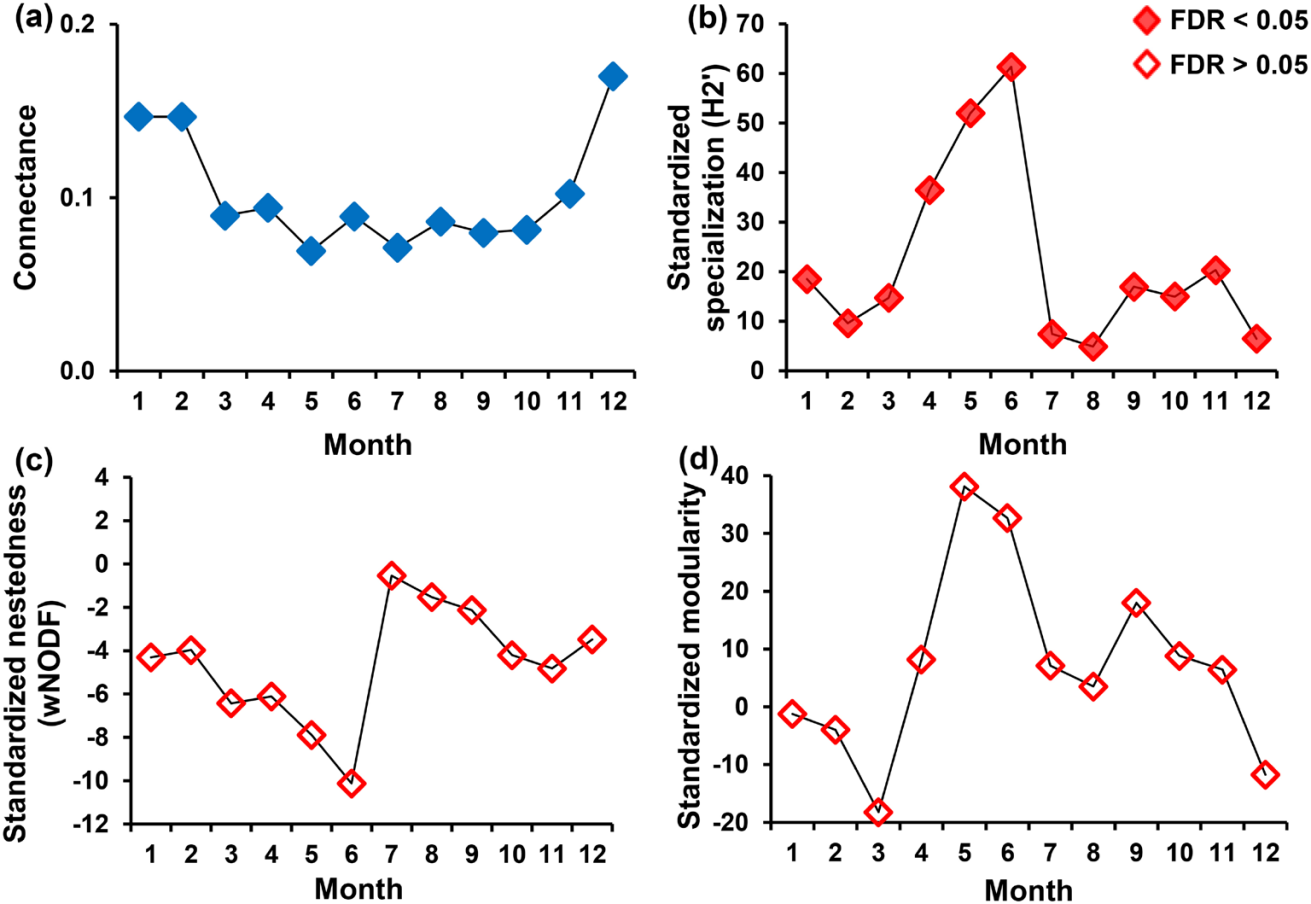
The dynamics of plant‐pollinator network structures over 12 months at the SCBG. (a) Connectance of the network. (b) Interaction specialization at the network level, measured by the H2′ index. (c) The weighted NODF index indicates network nestedness. (d) Barber's modularity index represents network modularity. The values for H2′, weighted NODF, and Barber's modularity indices are standardized based on randomization analysis. Positive values suggest that the network index estimates derived from the observed plant‐pollinator interaction matrices are greater than those of randomized matrices, while negative values indicate the opposite.

The plant‐pollinator network structure exhibited significant temporal variations (Figure 2 and Figure S3). From March to October, the network connectance remained below 0.1, while it increased during the winter months (November to February; Figure 3a). The network specialization was significantly higher than expected by chance throughout 12 months, with a notable increase from April to June (Figure 3b). Conversely, the network nestedness was significantly lower than expected by chance across 12 months, with an increase observed in July and August (Figure 3c). The network modularity was significantly higher than expected by chance from April to November, while lower in the remaining months. Networks exhibiting significant modularity, characterized by distinct compartmentalization into several modules of closely interacting plant and pollinator species, were observed from April to November.

**FIGURE 4:**
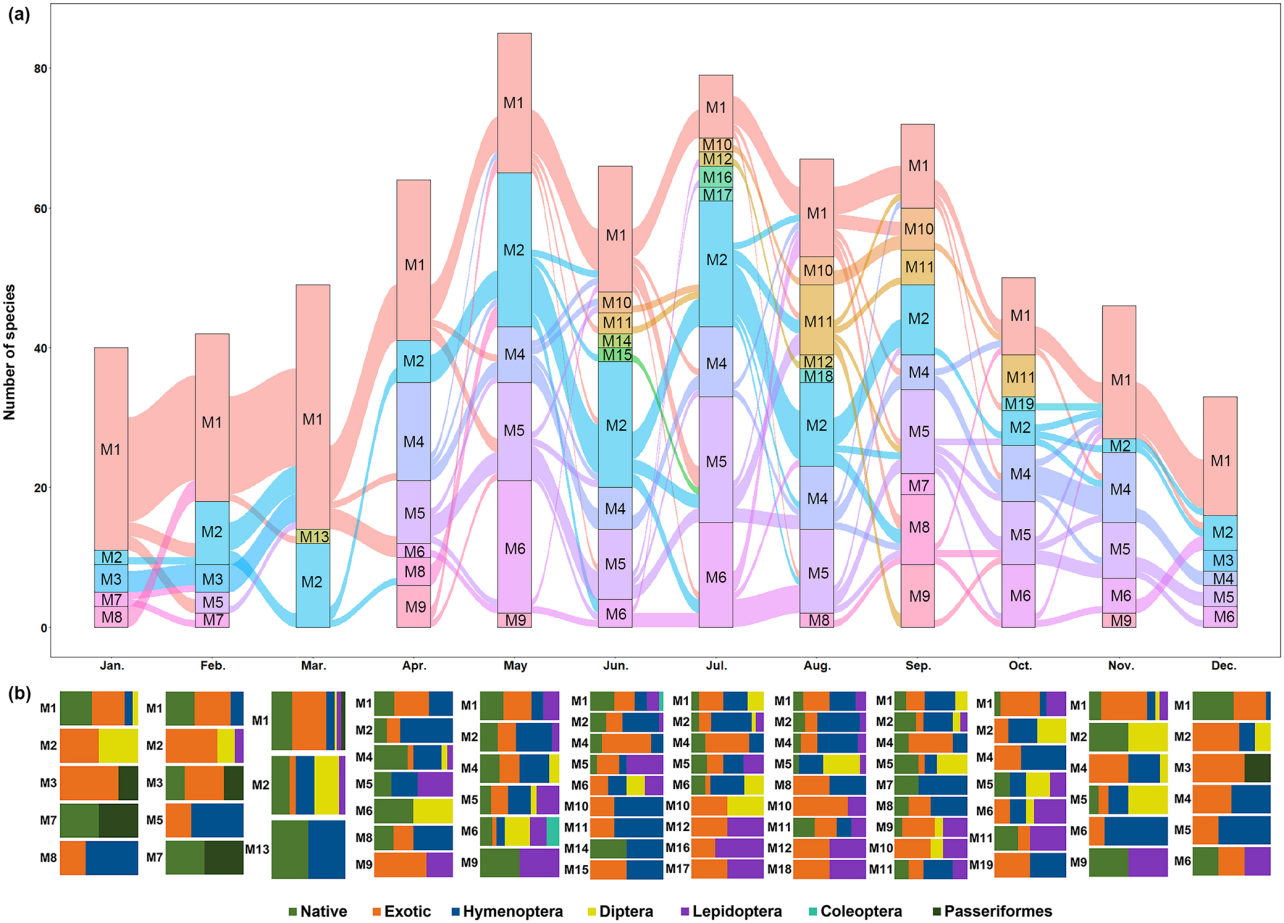
Succession of plant‐pollinator network modules through 12 months in the SCBG. (a) This alluvial diagram illustrates the flow of plant and pollinator species across different modules over 12 months, demonstrating how species migrate between modules throughout the year. The colored blocks represent clusters of species within modules, with their size proportional to the number of species contained therein. Module size is proportional to the total number of plant and pollinator species included. (b) Properties of network modules. For each network module identified in each month, the composition of plant species (categorized as native or exotic) and pollinator species (classified at the order level) is presented.

The results suggested that interactions between plants and pollinators changed dynamically over time (Figure 4a). Network modules identified in respective months changed considerably in their compositions of plant and pollinator species (Figure 4b). In April, for example, module 1 was dominated by exotic plants and Hymenoptera, while module 5 included only native plants and high proportions of Lepidoptera. After the fission of module 1 from March to April, in which exotic plants and Hymenoptera were abundant in March, the divisive module 6 in April included only native plants and Diptera. Following the fusion of module 2, characterized by native plants and Diptera, and module 6, featuring exotic plants and Hymenoptera, in November, the merged module (module 2) included exotic plants, Hymenoptera, and Diptera. The module dominated by Hymenoptera (module 2) was divided into three main modules differing in pollinator species in the transition from June to July and from July to August (Figure 4b).

### 3.4 Changes of Core Species

**FIGURE 5:**
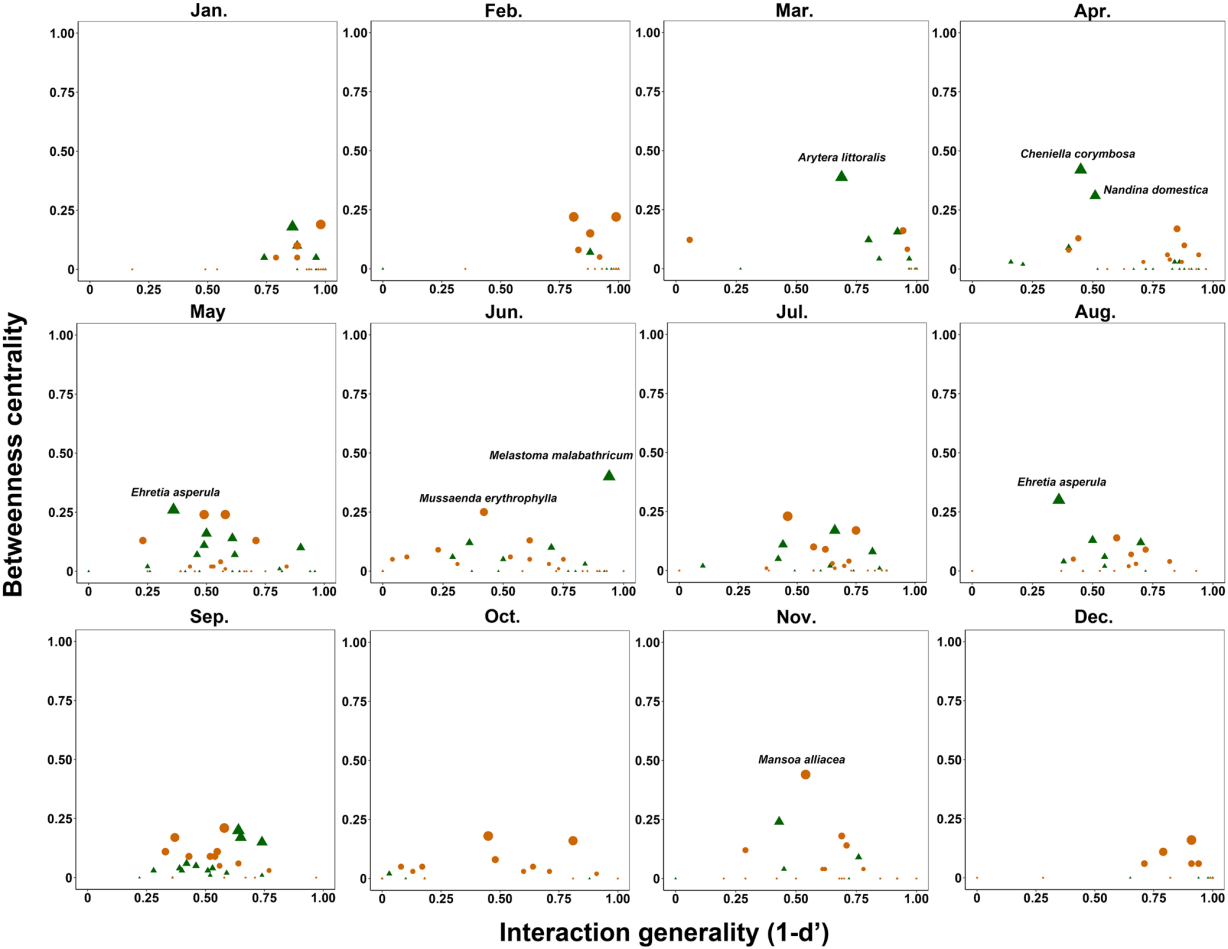
Monthly alternations of core species in plant‐pollinator networks in the SCBG. The interaction generality (1–*d*′) and betweenness centrality of each native and exotic plant species are shown on the horizontal and vertical axes, respectively. A high betweenness centrality value indicates that a species occupies a central position, connecting pairs of other species within the network. Plant species with a betweenness centrality > 0.25 are labeled with their names. Green triangles represent native plant species, while orange circles represent exotic plant species.

Our analysis revealed that the plant species having the highest values of interaction generality and betweenness centrality varied through 12 months (Figure 5 and Figure S4). Native and exotic plants demonstrated high interaction generality primarily during the winter months (December and from January to March). Six native plant species (within March, April, May, June, and August) and two exotic plant species (within June and November) exhibited betweenness centrality values exceeding 0.25 (Figure S4). Specifically, the native plant *Melastoma malabathricum* displayed the highest interaction generality and betweenness centrality in June (Figure 5). Another native plant, *Ehretia asperula*, had the highest betweenness centrality but moderate interaction generality in May and August (Figure 5 and Figure S4). The native plants *Arytera littoralis* in March and *Cheniella corymbosa* and *Nandina domestica* in April showed the highest betweenness centrality but moderate interaction generality (Figure 5 and Figure S4). In November, the exotic plant *Mansoa alliacea* exhibited the highest betweenness centrality within the community (Figure 5 and Figure S4). However, in the remaining six months, the highest betweenness scores of plant species were consistently below 0.25. In January, February, and December, the plant species displayed high interaction generality, while in July, September, and October, the plant species showed moderate interaction generality (Figure 5).

### 3.5 Core Species Across the Year

**FIGURE 6:**
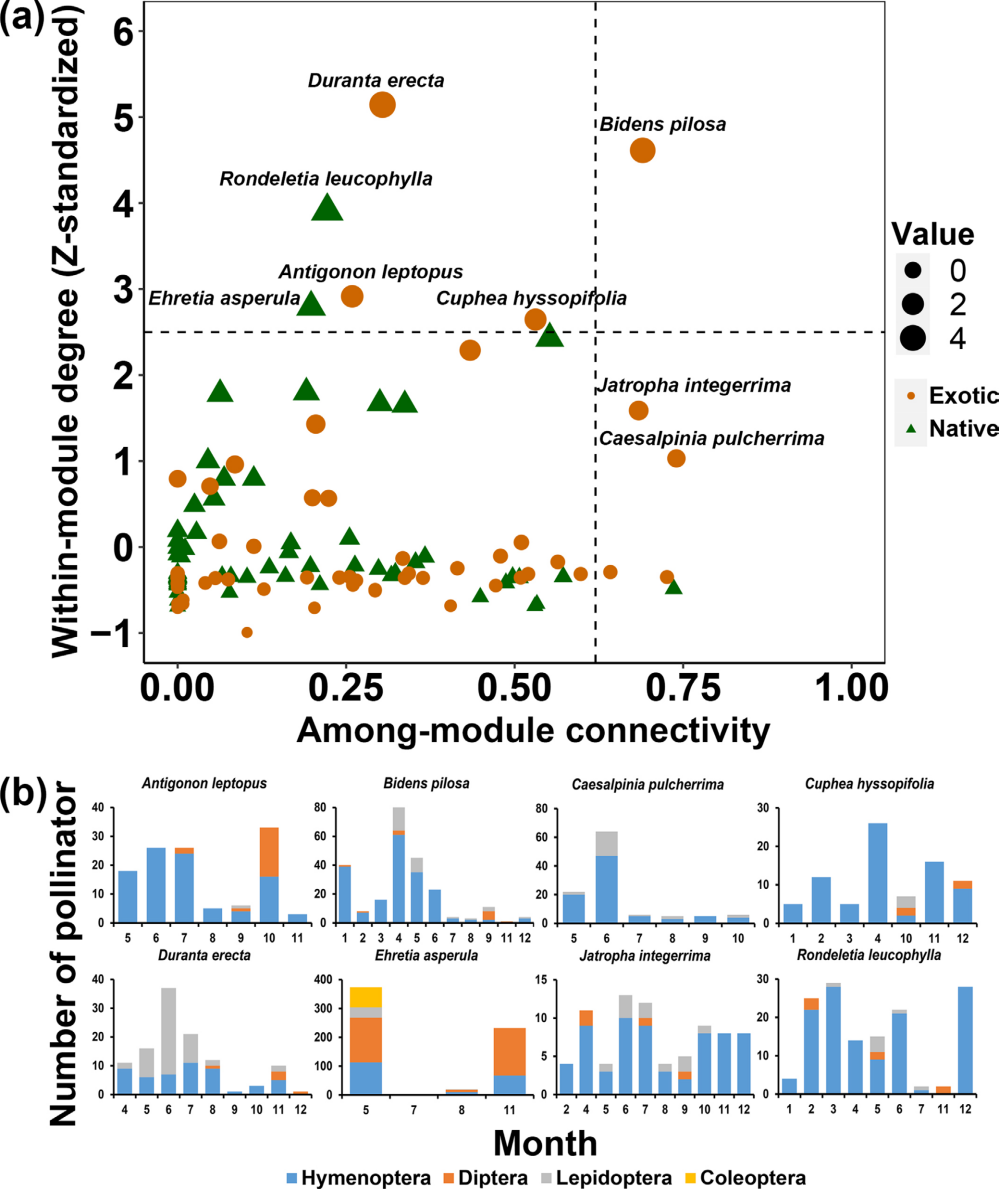
Core species in the meta‐network in the SCBG. (a) Plant species' topological roles within the meta‐network. For each native and exotic plant species in the meta‐network, among‐module connectivity and within‐module degree are shown along the horizontal and vertical axes, respectively. The lines indicate thresholds for defining ‘super generalists’, ‘connectors’, ‘module hubs’, and ‘peripherals’ in networks. (b) Temporal dynamics of pollinator species composition for plant species that exhibited high within‐module degree and among‐module connectivity (a).

The analysis of network modularity suggested that the meta‐network was classified into eight modules (Figure S5). Results suggested that the exotic plant species *Bidens pilosa* , pollinated by diverse pollinators, exhibited a high within‐module degree value. It also occupied positions that interlinked numerous modules in the meta‐network, which was evidenced by its high among‐module connectivity value (Figure 6a). After categorizing vertices within the network using these two indices based on the aforementioned criteria, two native and four exotic plant species were labeled as ‘super generalists’ or ‘module hubs’, while one native and four exotic plant species were classified as ‘connectors’ (Figure 6a). The native plant *Rondeletia leucophylla* and six exotic plants— *Antigonon leptopus* , *Bidens pilosa* , *Caesalpinia pulcherrima*, *Cuphea hyssopifolia* , *Duranta erecta* , and *Jatropha integerrima* —were characterized by their long‐term interactions with pollinators in the pollination network across years, with their pollinator composition varying among months (Figure 6b). The exotic plant *Bidens pilosa* was pollinated mainly by Hymenoptera from January to March and June to August, attracted Lepidoptera in April and May, and had Diptera as a dominant pollinator in September. It was designated as a super generalist based on its high within‐module degree (value > 2.5) and high among‐module connectivity (value > 0.62) (Figure 6b). In contrast, the native plants *Ehretia asperula* and *Rondeletia leucophylla*, along with the exotic plants *Antigonon leptopus* , *Cuphea hyssopifolia* , and *Duranta erecta* , were designated as super generalists but did not serve as connectors within the meta‐network (among‐module connectivity < 0.62) (Figure 6b). The two exotic plants, *Caesalpinia pulcherrima* and *Jatropha integerrima,* acted as connectors among modules while exhibiting low within‐module degree values (< 2.5) (Figure 6b).

## 4 Discussion

Our results suggested that plant‐pollinator interactions in SCBG exhibit high variability in both species composition and interaction patterns throughout one year. This finding confirms the importance of the temporal scale for understanding the structure of the plant‐pollinator interaction network (Petanidou et al. 2008; Burkle and Alarcón 2011). Our analysis provides a detailed and dynamic dataset of interactions between plants and pollinators in the SCBG over one year. The monthly turnover of native and exotic plants was identified as the primary driver of the monthly turnover of plant‐pollinator interactions. This result aligns with previous studies by Encinas‐Viso et al. (2012), Wang et al. (2020), Wang et al. (2021), and Hervías‐Parejo et al. (2023), which highlights the importance of plant species composition in explaining the dynamic variation of plant‐pollinator interactions. Plants and pollinators are more likely to co‐occur consistently over shorter timespans, such as days or weeks, compared to months. Consequently, interaction rewiring may become the main driver of the total beta‐diversity of interactions when data are collected at a finer temporal resolution.

Analyses of module affiliation in this study revealed monthly variation of the plant–pollinator networks. Network modules, consisting of closely interacting plant and pollinator species, constantly undergo fission and fusion across 12 months, illustrating the complex dynamics of interactions between plants and pollinators in the SCBG. These modules within the networks exhibited differences in their plant and pollinator species compositions, suggesting that pollinators of long‐flowering plants shift throughout the year. The data from this study, with species‐level resolution, offer a platform for understanding the specific dynamics of plant and pollinator interactions in SCBG. In the module composition, both native and exotic plants and pollinators of various functional groups, such as Diptera, Hymenoptera, and Lepidoptera, were grouped, suggesting that the origin of plant species and pollinator attributes was not a significant driver of plant–pollinator networks. The plant species turnover and the rewiring of interactions between long‐flowering plants and pollinators inevitably affect the variation in module composition and influence the structure of plant–pollinator networks across the year in SCBG.

Time series data of plant‐pollinator networks provide opportunities to reveal the variation of native plant and exotic plant species located at the core position of plant‐pollinator networks in the SCBG. The results showed that the core species within the plant‐pollinator networks in SCBG changed between native plants and exotic plants based on normalized betweenness values, with native plant species occupying the core mainly in spring and summer, while exotic plant species occupied the core mainly in winter. These findings indicate that the core plant species in plant‐pollinator networks and the potential influence of individual species on the whole community processes cannot be inferred without determining time scales (Suzuki et al. 2023). Consistent with the conclusions of previous research (Harrison and Winfree 2015; Staab et al. 2020; Zaninotto et al. 2023), the plants can be core species independently of their origin in plant‐pollinator networks, although this position varies throughout time. Exotic plants have been reported to be completely integrated within and drive the temporal dynamics of local plant‐pollinator networks (Valdovinos et al. 2009; Buchholz and Kowarik 2019; Staab et al. 2020; Wang et al. 2023; Zaninotto et al. 2023). The pollinators usually have no preferences between native plants and exotic plants in numerous urban green spaces (Nascimento et al. 2020; Staab et al. 2020), and thus both exotic and native plants have the opportunity to support and structure plant‐pollinator networks (Kovács‐Hostyánszki et al. 2022; Deshpande et al. 2023; Sánchez and Lara 2024). Therefore, both native plants and exotic plants have the opportunity to be core species in plant‐pollinator networks. This study highlights that there may be no one more important than the other between native plants and exotic plants in dynamic plant‐pollinator interaction networks. The roles of native plants and exotic plants in plant‐pollinator networks can only be well understood from a specific time viewpoint. These results emphasize that the role of a plant species within a plant‐pollinator network is more important than its origin in planning biodiversity‐friendly cities. In SCBG, generalist pollinators such as *Apis cerana* are most common, and they have no behavioral preferences for native or exotic plants. Studies have reported that generalist pollinators visit flowers of numerous plant species regardless of their geographic origin (Harrison and Winfree 2015; Wenzel et al. 2020; Berthon et al. 2021). Therefore, we should always consider the roles played by both native plants and exotic plants for urban sustainability when botanical garden greening in urban areas. Overall, these results propose that the management strategy should pay specific attention to the dynamic variation of core species in plant‐pollinator networks at the timescale. Feedback between observational and experimental studies is essential for fully understanding the dynamic nature of the roles of plant species in communities. For example, excluding target plant species at different times can enhance the understanding of the temporally varying roles of core species in the community.

The meta‐network analysis of core species revealed that each species was anticipated to interact with various pollinators at specific times throughout the year. The ‘module hubs’ and ‘connectors’ species in the meta‐network were all long‐flowering plants, interacting with diverse pollinators in different months at the SCBG. The results of species roles within and across modules demonstrated that both native and exotic plant species can serve as module hubs. Our findings support Arroyo‐Correa et al. (2020), indicating that exotic plant species interact with different pollinators throughout the year due to their longer flowering periods, making them vital connectors in the temporal networks. Exotic plants occupied core positions within the meta‐network, consistent with observations of invasive species (Larson et al. 2016; Russo et al. 2019; Wang et al. 2023). One Asteraceae species, *Bidens pilosa,* was the sole plant species that played a crucial role both within and among network modules, highlighting its significance in the SCBG community. Asteraceae species are generalists that attract various pollinators in different seasons, ultimately influencing the structure of plant‐pollinator networks (Lopezaraiza‐Mikel et al. 2007; Bartomeus et al. 2008; Wang et al. 2023). Therefore, when planning a strategy to increase food sources for insect pollinators in urban botanical gardens, priority should be given to ornamental plant species with long flowering periods spanning several months. Based on our findings, when assessing the roles of native plants and exotic plants within urban plant‐pollinator networks, the conservation plans are best to first think about the possible influences of gathering plant‐pollinator interactions data with the underlying temporal structures.

## 5 Conclusions

Keystone species are typically those species that exert excessively great ecological impacts on the surrounding biotic or abiotic environment about their abundance (Koski et al. 2015). In this context, network indices such as the standardized among‐module connectivity, which infers species impacts on network structure, can aid in identifying potential keystone species. The monthly time series network analysis conducted here may be expanded in some other directions. Even though species turnover across months can have a baseline effect, variations in interactions between species may be a major factor driving this succession. Investigating pollinator changes of plant species with extended flowering periods across months is fascinating in terms of the underlying mechanism by which the plasticity of plant‐pollinator networks contributes to community stability and sustainability. Further research is warranted to explore the processes behind the succession, rules, and influencing factors of core plant species between native and exotic plant species within plant‐pollinator networks. Our current analysis included only one year of plant‐pollinator interaction data collected at a single botanical garden, reflecting a single year's phenomenon. However, it is crucial to explore inter‐annual and among‐garden variation in dynamic plant‐pollinator interaction network structures. Time series data of plants and pollinators' interactions can improve and deepen our understanding of the stability of ecosystem processes.

## Author Contributions

**Xiang‐Ping Wang:** conceptualization (equal), data curation (lead), formal analysis (lead), funding acquisition (lead), investigation (equal), methodology (lead), visualization (lead), writing – original draft (lead), writing – review and editing (lead). **Shi‐Ran Gu:** investigation (equal), methodology (equal). **Zhong‐Tao Zhao:** methodology (equal), writing – original draft (equal). **Shi‐Jin Li:** writing – original draft (equal). **Miao‐Miao Shi:** conceptualization (equal), methodology (equal), writing – original draft (equal), writing – review and editing (equal). **Tie‐Yao Tu:** conceptualization (equal), methodology (equal), writing – review and editing (equal).

## Conflicts of Interest

The authors declare no conflicts of interest.

## Supporting information


**FIGURE S1.** Comparison of network modularity algorithms. For the calculation of network modularity, three module‐detecting algorithms, including ‘Louvain’, ‘fast greedy’, and ‘Infomap’, were compared. Higher values of modularity represent more optimized module memberships.

**FIGURE S2.** Native and exotic plant and pollinator species compositions across 12 months in the SCBG. The figure displays the number of native and exotic plant species alongside the number of pollinator species observed in each month.

**FIGURE S3.** Plant‐pollinator network modules for each month in the SCBG. For each plant‐pollinator interaction network, closely interacting plant and pollinator species were classified into the same modules based on the ‘Louvain’ algorithm. Colors represent modules detected in each network.

**FIGURE S4.** The normalized betweenness centrality values of native and exotic plant species in each month in the SCBG. Plant species with betweenness centrality values < 0.1 in each network were not shown.

**FIGURE S5.** Meta‐network depicting all plant‐pollinator interactions recorded from January to December in the SCBG. Colors represent modules detected within the meta‐network.

**TABLE S1.** Plant species codes used in this research.

**TABLE S2.** Pollinator species codes used in this research. The identification method for each pollinator species were shown (Morphological ID: Morphological identification; Molecular ID: Molecular identification).

## Data Availability

All supplementary data needed to evaluate the conclusions of the paper are in the paper and/or supporting information (Tables S1 and S2). Additional data relevant to this paper that support the findings of this study are available from the corresponding author upon reasonable request.
