# Supplementary figures and images for: Monthly Alternations of Core Plant Species in Dynamic Plant‐Pollinator Networks of an Urban Botanical Garden
Source: Ecol Evol. 2025 Jul 17;15(7):e71822. doi: 10.1002/ece3.71822 (PMC12270637; doi:10.1002/ece3.71822)

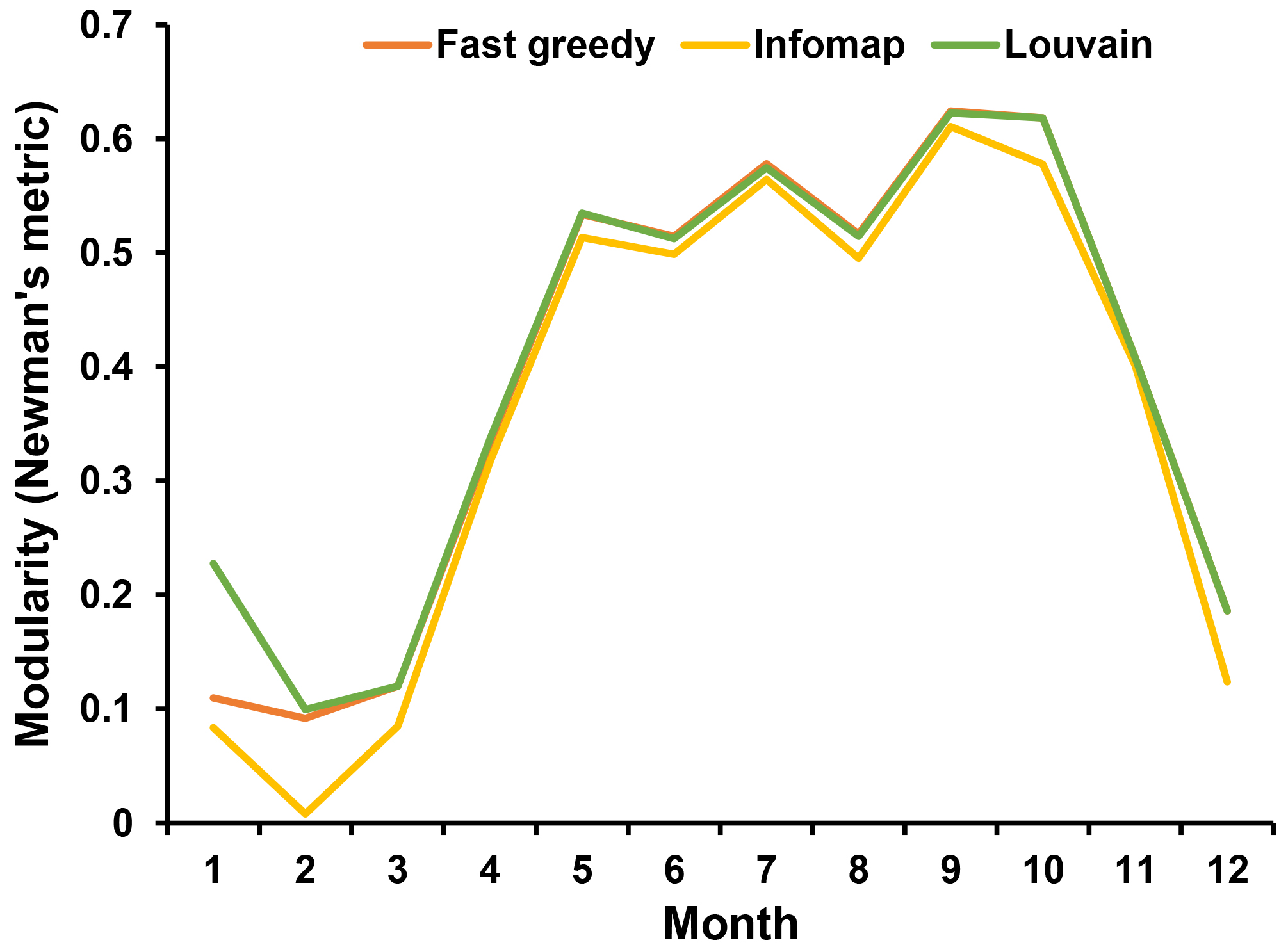

Supplement: Supplementary file 1 — FIGURE S1. Comparison of network modularity algorithms. For the calculation of network modularity, three module‐detecting algorithms, including ‘Louvain’, ‘fast greedy’, and ‘Infomap’, were compared. Higher values of modularity represent more optimized module memberships. [file ECE3-15-e71822-s003.jpg]

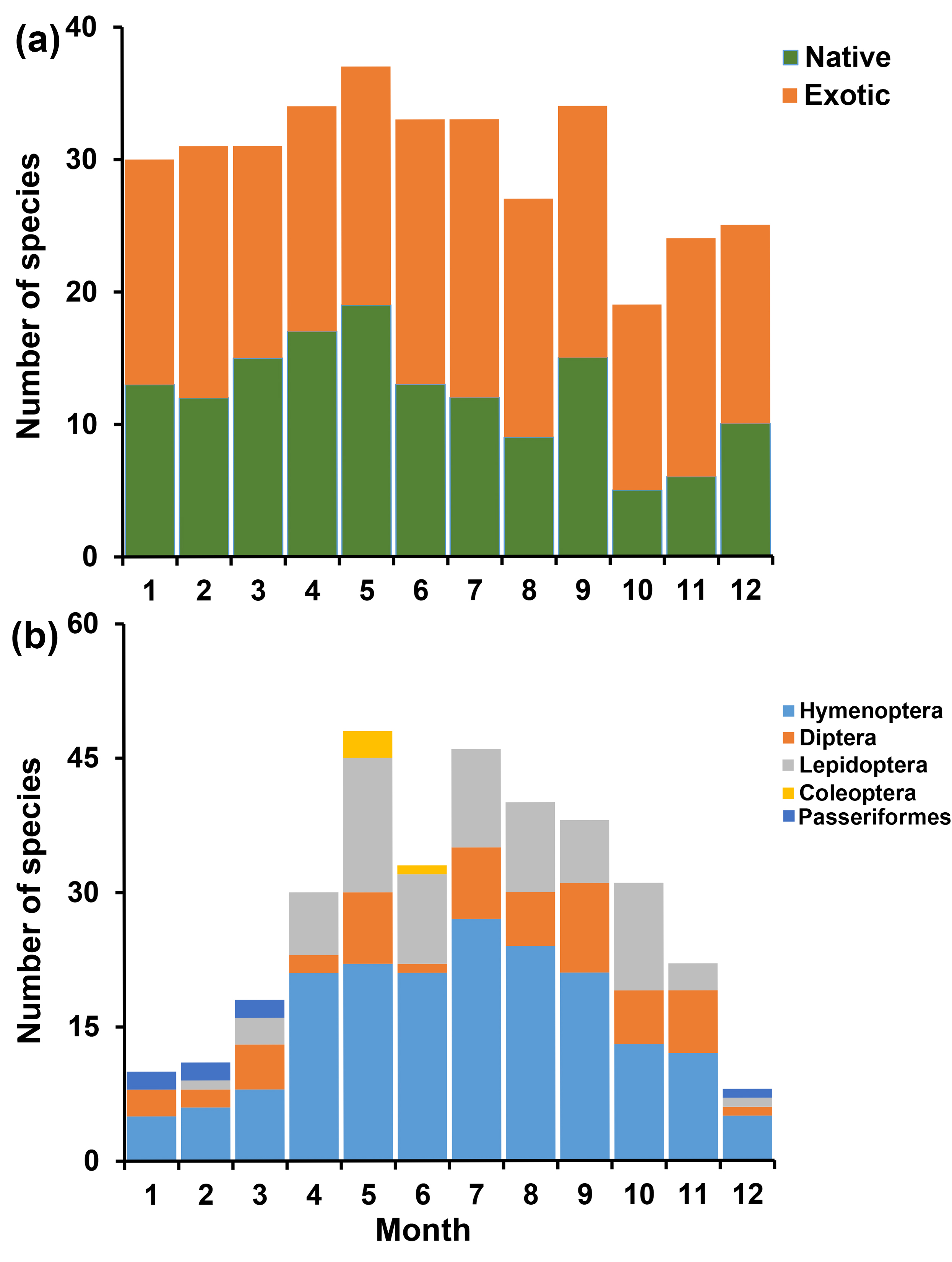

Supplement: Supplementary file 2 — FIGURE S2. Native and exotic plant and pollinator species compositions across 12 months in the SCBG. The figure displays the number of native and exotic plant species alongside the number of pollinator species observed in each month. [file ECE3-15-e71822-s002.jpg]

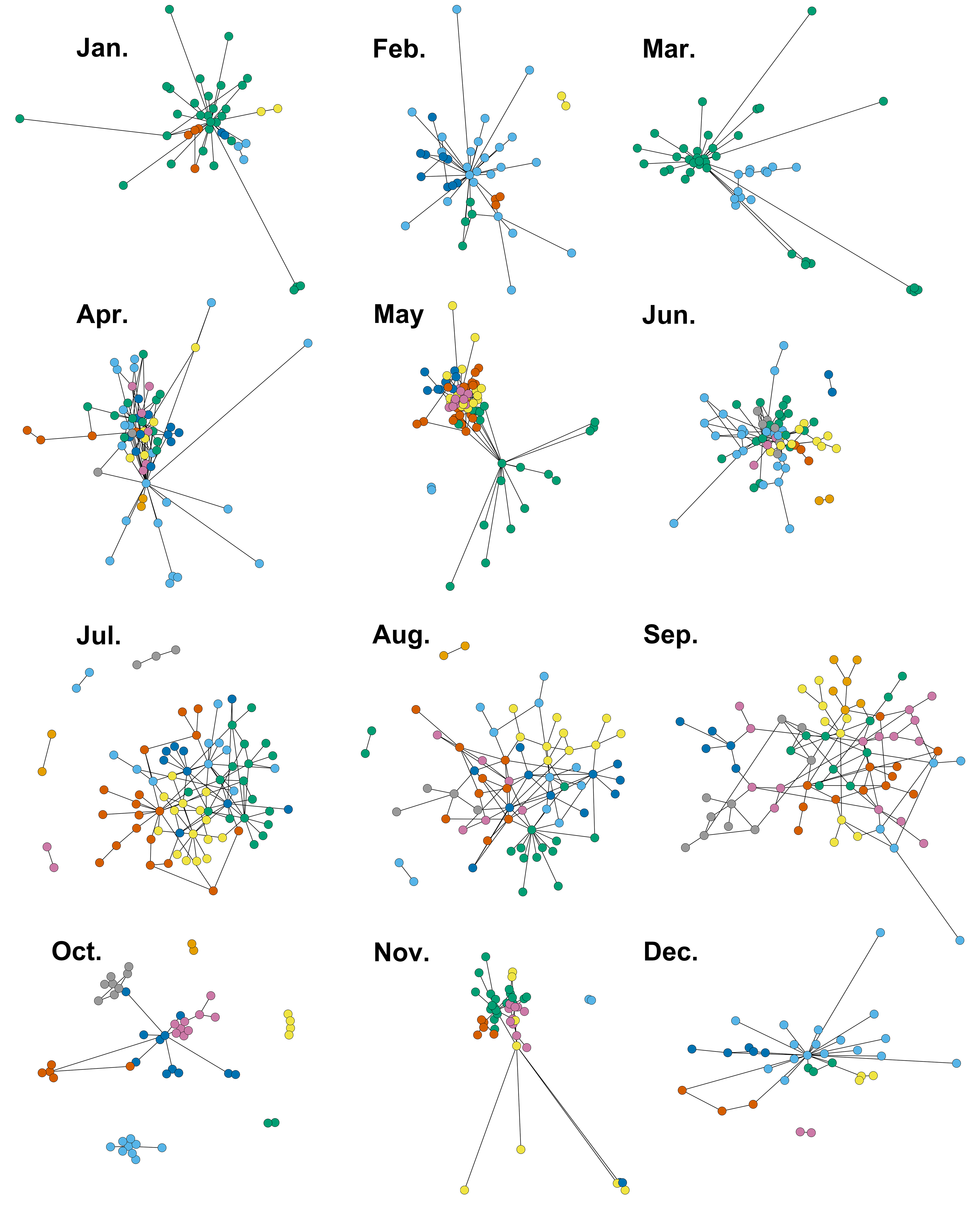

Supplement: Supplementary file 3 — FIGURE S3. Plant‐pollinator network modules for each month in the SCBG. For each plant‐pollinator interaction network, closely interacting plant and pollinator species were classified into the same modules based on the ‘Louvain’ algorithm. Colors represent modules detected in each network. [file ECE3-15-e71822-s005.jpg]

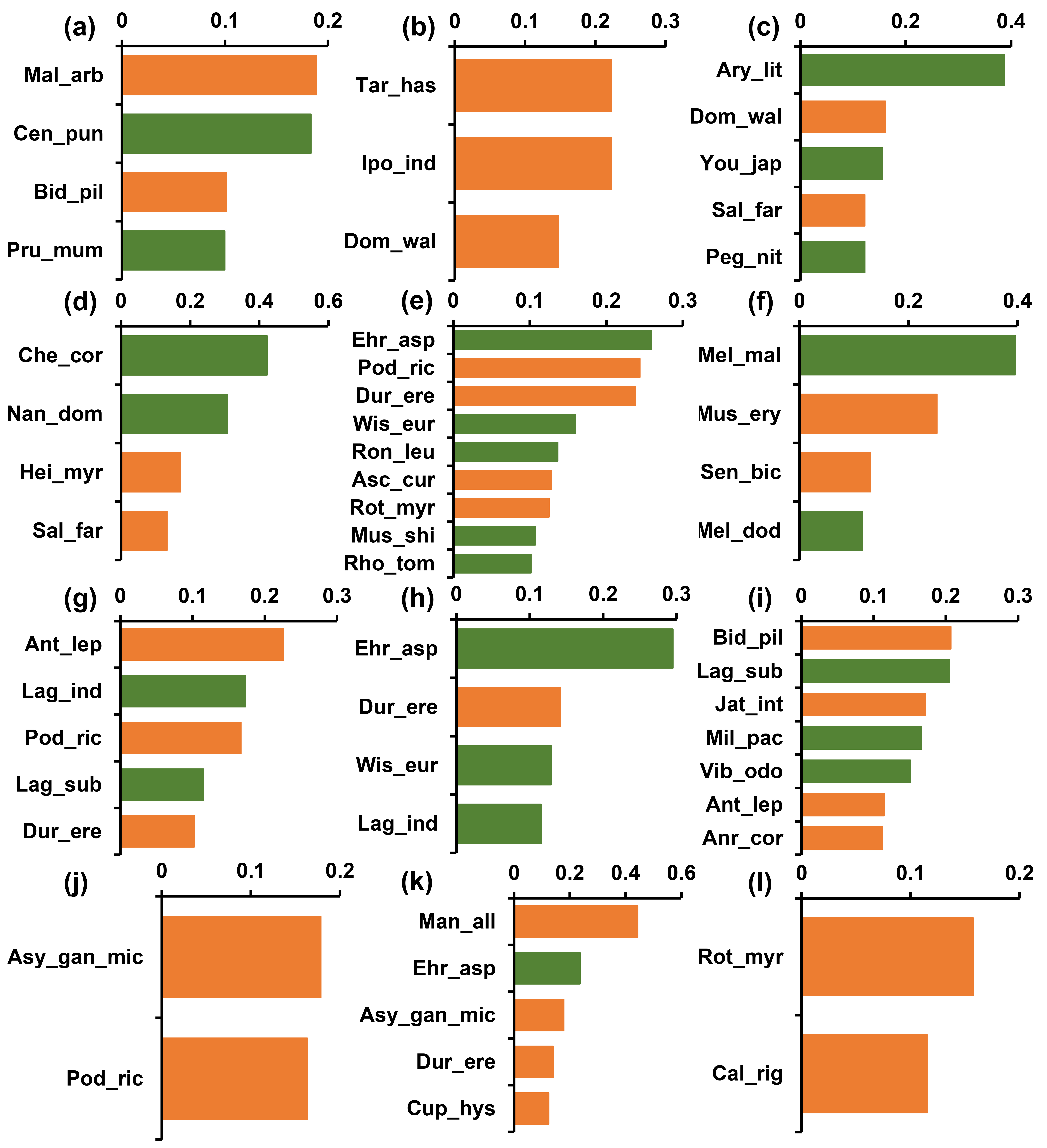

Supplement: Supplementary file 4 — FIGURE S4. The normalized betweenness centrality values of native and exotic plant species in each month in the SCBG. Plant species with betweenness centrality values < 0.1 in each network were not shown. [file ECE3-15-e71822-s004.jpg]

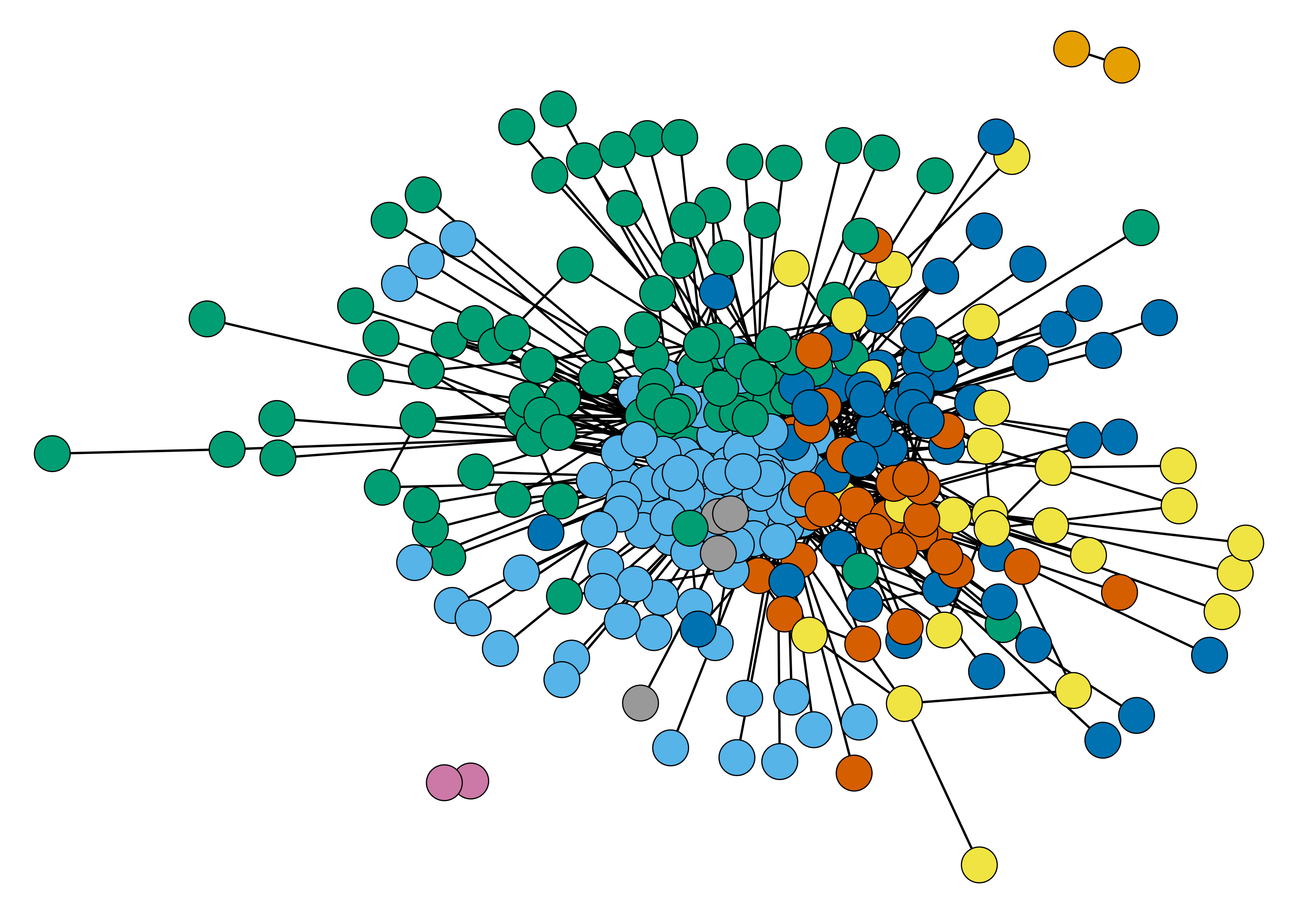

Supplement: Supplementary file 5 — FIGURE S5. Meta‐network depicting all plant‐pollinator interactions recorded from January to December in the SCBG. Colors represent modules detected within the meta‐network. [file ECE3-15-e71822-s006.jpg]
