# Supplementary material for: Monthly Alternations of Core Plant Species in Dynamic Plant‐Pollinator Networks of an Urban Botanical Garden
Source: Ecol Evol. 2025 Jul 17;15(7):e71822. doi: 10.1002/ece3.71822 (PMC12270637; doi:10.1002/ece3.71822)
Supplement: Supplementary file 6 — TABLE S1. Plant species codes used in this research. [file ECE3-15-e71822-s001.docx]

**Table S1**. Plant species codes used in this research.

| **Code** | **Species** | **Family** |
| --- | --- | --- |
| Abu_pic | *Abutilon pictum* | Malvaceae |
| Aca_pod | *Acacia podalyriifolia* | Fabaceae |
| Act_arg | *Actinidia arguta* | Actinidiaceae |
| Act_lat | *Actinidia latifolia* | Actinidiaceae |
| Act_mac | *Actinidia macrosperma* | Actinidiaceae |
| Aga_afr | *Agapanthus africanus* | Amaryllidaceae |
| All_cat | *Allamanda cathartica* | Apocynaceae |
| Alp_hai | *Alpinia hainanensis* | Zingiberaceae |
| Amp_gla | *Ampelopsis glandulosa* | Vitaceae |
| Anr_cor | *Anredera cordifolia* | Basellaceae |
| Ant_lep | *Antigonon leptopus* | Polygonaceae |
| Ant_maj | *Antirrhinum majus* | Plantaginaceae |
| Aqu_gla | *Aquilegia glandulosa* | Ranunculaceae |
| Ara_dur | *Arachis duranensis* | Fabaceae |
| Ard_den | *Ardisia densilepidotula* | Primulaceae |
| Arg_cap | *Argyreia capitiformis* | Convolvulaceae |
| Ary_lit | *Arytera littoralis* | Sapindaceae |
| Asc_cur | *Asclepias curassavica* | Apocynaceae |
| Asy_gan | *Asystasia gangetica* | Acanthaceae |
| Asy_gan_mic | *Asystasia gangetica subsp. micrantha* | Acanthaceae |
| Ave_car | *Averrhoa carambola* | Oxalidaceae |
| Bar_cri | *Barleria cristata* | Acanthaceae |
| Bau_bla | *Bauhinia blakeana* | Fabaceae |
| Bid_pil | *Bidens pilosa* | Asteraceae |
| Bou_spe | *Bougainvillea spectabilis* | Nyctaginaceae |
| Bri_owa | *Brillantaisia owariensis* | Acanthaceae |
| Bru_bra | *Brunfelsia brasiliensis* | Solanaceae |
| Bru_pau | *Brunfelsia pauciflora* | Solanaceae |
| Bud_fal | *Buddleja fallowiana* | Scrophulariaceae |
| Bul_fru | *Bulbine frutescens* | Asphodelaceae |
| Cae_pul | *Caesalpinia pulcherrima* | Fabaceae |
| Cal_nit | *Callerya nitida* | Fabaceae |
| Cal_sur | *Calliandra surinamensis* | Fabaceae |
| Cal_hae | *Calliandra haematocephala* | Fabaceae |
| Cal_rig | *Callistemon rigidus* | Myrtaceae |
| Cal_vim | *Callistemon viminalis* | Myrtaceae |
| Cam_gra | *Campsis grandiflora* | Bignoniaceae |
| Can_ind | *Canna indica* | Cannaceae |
| Car_hal | *Cardiospermum halicacabum* | Sapindaceae |
| Cel_arg | *Celosia argentea* | Amaranthaceae |
| Cen_pun | *Centratherum punctatum* | Asteraceae |
| Cer_man | *Cerbera manghas* | Apocynaceae |
| Che_spe | *Cheilocostus speciosus* | Zingiberaceae |
| Che_cor | *Cheniella corymbosa* | Fabaceae |
| Che_gla | *Cheniella glauca* | Fabaceae |
| Che_tou | *Cheniella touranensis* | Fabaceae |
| Chr_odo | *Chrysojasminum odoratissimum* | Oleaceae |
| Cli_ter | *Clitoria ternatea* | Fabaceae |
| Coc_gra | *Coccinia grandis* | Cucurbitaceae |
| Cup_hys | *Cuphea hyssopifolia* | Lythraceae |
| Dah_pin | *Dahlia pinnata* | Asteraceae |
| Der_for | *Derris fordii* | Fabaceae |
| Dom_wal | *Dombeya wallichii* | Malvaceae |
| Dur_ere | *Duranta erecta* | Verbenaceae |
| Ehr_asp | *Ehretia asperula* | Boraginaceae |
| Ela_con | *Elaeagnus conferta* | Elaeagnaceae |
| Ela_pun | *Elaeagnus pungens* | Elaeagnaceae |
| Ela_hai | *Elaeocarpus hainanensis* | Elaeocarpaceae |
| Ela_rug | *Elaeocarpus rugosus* | Elaeocarpaceae |
| Era_pul | *Eranthemum pulchellum* | Acanthaceae |
| Ery_hai | *Erycibe hainanensis* | Convolvulaceae |
| Eur_pec | *Euryops pectinatus* | Asteraceae |
| Evo_nut | *Evolvulus nuttallianus* | Convolvulaceae |
| Fib_rec | *Fibraurea recisa* | Menispermaceae |
| Fuc_hyb | *Fuchsia hybrida* | Onagraceae |
| Gal_gra | *Galphimia gracilis* | Malpighiaceae |
| Gel_sem | *Gelsemium sempervirens* | Gelsemiaceae |
| Gom_glo | *Gomphrena globosa* | Amaranthaceae |
| Gro_het | *Grona heterocarpos* | Fabaceae |
| Han_chr | *Handroanthus chrysanthus* | Bignoniaceae |
| Han_imp | *Handroanthus impetiginosus* | Bignoniaceae |
| Hay_wal | *Haymondia wallichii* | Fabaceae |
| Hei_myr | *Heimia myrtifolia* | Lythraceae |
| Hib_gre | *Hibiscus grewiifolius* | Malvaceae |
| Hib_ham | *Hibiscus hamabo* | Malvaceae |
| Hib_mut | *Hibiscus mutabilis* | Malvaceae |
| Hib_ros | *Hibiscus rosa-sinensis* | Malvaceae |
| Hib_sch | *Hibiscus schizopetalus* | Malvaceae |
| Hyp_mon | *Hypericum monogynum* | Hypericaceae |
| Ipo_ind | *Ipomoea indica* | Convolvulaceae |
| Ixo_chi | *Ixora chinensis* | Rubiaceae |
| Jas_sam | *Jasminum sambac* | Oleaceae |
| Jas_elo | *Jasminum elongatum* | Oleaceae |
| Jat_int | *Jatropha integerrima* | Euphorbiaceae |
| Lag_ind | *Lagerstroemia indica* | Lythraceae |
| Lag_lim | *Lagerstroemia limii* | Lythraceae |
| Lag_spe | *Lagerstroemia speciosa* | Lythraceae |
| Lag_sub | *Lagerstroemia subcostata* | Lythraceae |
| Lew_cot | *Lewisia cotyledon* | Montiaceae |
| Mal_arb | *Malvaviscus arboreus* | Malvaceae |
| Mal_pen | *Malvaviscus penduliflorus* | Malvaceae |
| Man_all | *Mansoa alliacea* | Bignoniaceae |
| Mel_dod | *Melastoma dodecandrum* | Melastomataceae |
| Mel_mal | *Melastoma malabathricum* | Melastomataceae |
| Mel_mal_alb | *Melastoma malabathricum var. alba* | Melastomataceae |
| Mel_coc | *Melodinus cochinchinensis* | Apocynaceae |
| Mil_pac | *Millettia pachyloba* | Fabaceae |
| Mom_coc | *Momordica cochinchinensis* | Cucurbitaceae |
| Mun_cal | *Muntingia calabura* | Muntingiaceae |
| Mur_exo | *Murraya exotica* | Rutaceae |
| Mus_Ali | *Mussaenda 'Alicia'* | Rubiaceae |
| Mus_ery | *Mussaenda erythrophylla* | Rubiaceae |
| Mus_shi | *Mussaenda shikokiana* | Rubiaceae |
| Nan_dom | *Nandina domestica* | Berberidaceae |
| Nek_gro | *Nekemias grossedentata* | Vitaceae |
| Neo_gra | *Neomarica gracilis* | Iridaceae |
| Och_ser | *Ochna serrulata* | Ochnaceae |
| Odo_cal | *Odontonema callistachyum* | Acanthaceae |
| Pae_cru | *Paederia cruddasiana* | Rubiaceae |
| Pas_cae | *Passiflora caerulea* | Passifloraceae |
| Peg_nit | *Pegia nitida* | Anacardiaceae |
| Per_hyb | *Pericallis hybrida* | Asteraceae |
| Pet_vol | *Petrea volubilis* | Verbenaceae |
| Pha_cha | *Phanera championii* | Fabaceae |
| Phl_cur | *Phlogacanthus curviflorus* | Acanthaceae |
| Pie_jap | *Pieris japonica* | Ericaceae |
| Plu_aur | *Plumbago auriculata* | Plumbaginaceae |
| Pod_ric | *Podranea ricasoliana* | Bignoniaceae |
| Pru_mum | *Prunus mume* | Rosaceae |
| Rha_ind | *Rhaphiolepis indica* | Rosaceae |
| Rho_pul | *Rhododendron pulchrum* | Ericaceae |
| Rho_sim | *Rhododendron simsii* | Ericaceae |
| Rho_tom | *Rhodomyrtus tomentosa* | Myrtaceae |
| Ron_leu | *Rondeletia leucophylla* | Rubiaceae |
| Ros_mul_Gre | *Rosa multiflora 'Grevillei'* | Rosaceae |
| Rot_myr | *Rotheca myricoides* | Lamiaceae |
| Rub_ros | *Rubus rosifolius* | Rosaceae |
| Rue_sim | *Ruellia simplex* | Acanthaceae |
| Sal_far | *Salvia farinacea* | Lamiaceae |
| Sal_leu | *Salvia leucantha* | Lamiaceae |
| Sal_spl | *Salvia splendens* | Lamiaceae |
| Sen_ala | *Senna alata* | Fabaceae |
| Sen_bic | *Senna bicapsularis* | Fabaceae |
| Sid_sub | *Sida subcordata* | Malvaceae |
| Sol_wri | *Solanum wrightii* | Solanaceae |
| Sph_tri | *Sphagneticola trilobata* | Asteraceae |
| Str_cus | *Strobilanthes cusia* | Acanthaceae |
| Str_ham | *Strobilanthes hamiltoniana* | Acanthaceae |
| Tar_has | *Tarenaya hassleriana* | Cleomaceae |
| Tec_cap | *Tecoma capensis* | Acanthaceae |
| Thu_ala | *Thunbergia alata* | Acanthaceae |
| Thu_lau | *Thunbergia laurifolia* | Acanthaceae |
| Tib_sem | *Tibouchina semidecandra* | Melastomataceae |
| Uva_gra | *Uvaria grandiflora* | Annonaceae |
| Vib_han | *Viburnum hanceanum* | Adoxaceae |
| Vib_odo | *Viburnum odoratissimum* | Adoxaceae |
| Wis_eur | *Wisteriopsis eurybotrya* | Fabaceae |
| Wis_ret | *Wisteriopsis reticulata* | Fabaceae |
| You_jap | *Youngia japonica* | Asteraceae |
| Zan_nit | *Zanthoxylum nitidum* | Rutaceae |
| Ziz_mau | *Ziziphus mauritiana* | Rhamnaceae |
