# Supplementary material for: Monthly Alternations of Core Plant Species in Dynamic Plant‐Pollinator Networks of an Urban Botanical Garden
Source: Ecol Evol. 2025 Jul 17;15(7):e71822. doi: 10.1002/ece3.71822 (PMC12270637; doi:10.1002/ece3.71822)
Supplement: Supplementary file 7 — TABLE S2. Pollinator species codes used in this research. The identification method for each pollinator species were shown (Morphological ID: Morphological identification; Molecular ID: Molecular identification). [file ECE3-15-e71822-s007.docx]

**Table S2.** Pollinator species codes used in this research. The identification method for each pollinator species were shown (Morphological ID: Morphological identification; Molecular ID: Molecular identification).

| **Code** | **Species** | **Taxa** | **ID method** |
| --- | --- | --- | --- |
| Aet_chr | *Aethopyga christinae* | Passeriformes | Morphological ID |
| All_chi | *Allorhynchium chinense* | Hymenoptera | Molecular ID |
| Ama_luc | *Amata lucerna* | Lepidoptera | Morphological ID |
| Ama_phe | *Amata phegea* | Lepidoptera | Molecular ID |
| Ama_spe | *Amata sperbius* | Lepidoptera | Morphological ID |
| Ame_cal | *Amegilla calceifera* | Hymenoptera | Molecular ID |
| Ame_tho | *Amegilla thorogoodi* | Hymenoptera | Molecular ID |
| Ant_abd | *Anterhynchium abdominale* | Hymenoptera | Molecular ID |
| Api_cer | *Apis cerana* | Hymenoptera | Morphological ID |
| Apo_tro | *Apodynerus troglodytes* | Hymenoptera | Molecular ID |
| Bac_dor | *Bactrocera dorsalis* | Diptera | Molecular ID |
| Bao_far | *Baoris farri* | Lepidoptera | Molecular ID |
| Bom_bic | *Bombus bicoloratus* | Hymenoptera | Molecular ID |
| Bor_cin | *Borbo cinnara* | Lepidoptera | Molecular ID |
| Bra_mix | *Braunsapis mixta* | Hymenoptera | Molecular ID |
| Cat_pom | *Catopsilia pomona* | Lepidoptera | Morphological ID |
| Cat_pyr | *Catopsilia pyranthe* | Lepidoptera | Morphological ID |
| Cep_ner | *Cepora nerissa* | Lepidoptera | Molecular ID |
| Cer_apa | *Ceratina apacheorum* | Hymenoptera | Molecular ID |
| Cer_bry | *Ceratina bryanti* | Hymenoptera | Molecular ID |
| Cer_den | *Ceratina dentipes* | Hymenoptera | Morphological ID |
| Cer_oki | *Ceratina okinawana* | Hymenoptera | Morphological ID |
| Cer_sma | *Ceratina smaragdula* | Hymenoptera | Morphological ID |
| Cer_sut | *Ceratina sutepensis* | Hymenoptera | Molecular ID |
| Cer_hor | *Cerceris hortivaga* | Hymenoptera | Molecular ID |
| Cer_ryb | *Cerceris rybyensis* | Hymenoptera | Molecular ID |
| Cet_pil | *Cetonia pilifera* | Coleoptera | Morphological ID |
| Chi_pan | *Chilades pandava* | Lepidoptera | Molecular ID |
| Chl_mac | *Chlorophorus macaumensis* | Coleoptera | Morphological ID |
| Cte_els | *Ctenoplectra elsei* | Hymenoptera | Molecular ID |
| Cup_ery | *Cupha erymanthis* | Lepidoptera | Morphological ID |
| Dan_chr | *Danaus chrysippus* | Lepidoptera | Morphological ID |
| Did_aeg | *Dideopsis aegrota* | Diptera | Molecular ID |
| Ect_con | *Ectemnius continuus* | Hymenoptera | Molecular ID |
| Ect_tri | *Ectemnius trifasciatus* | Hymenoptera | Molecular ID |
| Ely_hyp | *Elymnias hypermnestra* | Lepidoptera | Molecular ID |
| Epi_bal | *Episyrphus balteatus* | Diptera | Molecular ID |
| Eri_arv | *Eristalinus arvorum* | Diptera | Morphological ID |
| Eri_qui | *Eristalinus quinquestriatus* | Diptera | Molecular ID |
| Eri_sur | *Eristalinus surcoufi* | Diptera | Molecular ID |
| Eri_tar | *Eristalinus tarsalis* | Diptera | Molecular ID |
| Eri_rup | *Eristalis rupium* | Diptera | Molecular ID |
| Eri_ten | *Eristalis tenax* | Diptera | Molecular ID |
| Euc_cne | *Euchrysops cnejus* | Lepidoptera | Molecular ID |
| Eum_inc | *Eumenes inconspicuus* | Hymenoptera | Molecular ID |
| Eup_mid | *Euploea midamus* | Lepidoptera | Morphological ID |
| Exo_cap | *Exoprosopa capucina* | Diptera | Molecular ID |
| Gra_aga | *Graphium agamemnon* | Lepidoptera | Morphological ID |
| Gra_eur | *Graphium eurypylus* | Lepidoptera | Morphological ID |
| Gra_sar | *Graphium sarpedon* | Lepidoptera | Morphological ID |
| Gra_sem | *Graptomyza semicircularia* | Diptera | Molecular ID |
| Hed_ger | *Hedychrum gerstaeckeri* | Hymenoptera | Molecular ID |
| Hel_aff | *Helophilus affinis* | Diptera | Molecular ID |
| Hem_lig | *Hemipyrellia ligurriens* | Diptera | Morphological ID |
| Her_lea | *Heriades leavitti* | Hymenoptera | Molecular ID |
| Hom_dam | *Homalictus dampieri* | Hymenoptera | Molecular ID |
| Hyl_ins | *Hylaeus insularum* | Hymenoptera | Molecular ID |
| Idi_div | *Idiella divisa* | Diptera | Molecular ID |
| Isc_scu | *Ischiodon scutellaris* | Diptera | Molecular ID |
| Jun_alm | *Junonia almana* | Lepidoptera | Morphological ID |
| Jun_iph | *Junonia iphita* | Lepidoptera | Molecular ID |
| Jun_lem | *Junonia lemonias* | Lepidoptera | Molecular ID |
| Lam_boe | *Lampides boeticus* | Lepidoptera | Molecular ID |
| Las_hof | *Lasioglossum hoffmanni* | Hymenoptera | Molecular ID |
| Las_nig | *Lasioglossum nigripes* | Hymenoptera | Molecular ID |
| Las_ser | *Lasioglossum serenum* | Hymenoptera | Molecular ID |
| Las_sex | *Lasioglossum sexstrigatum* | Hymenoptera | Molecular ID |
| Lia_ery | *Liacos erythrosoma* | Hymenoptera | Molecular ID |
| Luc_pap | *Lucilia papuensis* | Diptera | Molecular ID |
| Mac_pyr | *Macroglossum pyrrhostictum* | Lepidoptera | Morphological ID |
| Meg_pri | *Megacampsomeris prismatica* | Hymenoptera | Molecular ID |
| Meg_ang | *Megachile angustistrigata* | Hymenoptera | Molecular ID |
| Meg_cir | *Megachile circumcincta* | Hymenoptera | Molecular ID |
| Meg_con | *Megachile conjuncta* | Hymenoptera | Molecular ID |
| Meg_coq | *Megachile coquimbensis* | Hymenoptera | Molecular ID |
| Meg_dis | *Megachile disjuncta* | Hymenoptera | Molecular ID |
| Meg_disj | *Megachile disjunctiformis* | Hymenoptera | Molecular ID |
| Meg_fac | *Megachile faceta* | Hymenoptera | Molecular ID |
| Meg_fla | *Megachile flabellipes* | Hymenoptera | Molecular ID |
| Meg_her | *Megachile hera* | Hymenoptera | Molecular ID |
| Meg_ler | *Megachile lerma* | Hymenoptera | Molecular ID |
| Meg_ram | *Megachile ramakrishnae* | Hymenoptera | Molecular ID |
| Meg_sau | *Megachile saulcyi* | Hymenoptera | Molecular ID |
| Meg_spi | *Megachile spissula* | Hymenoptera | Molecular ID |
| Meg_str | *Megachile strupigera* | Hymenoptera | Molecular ID |
| Meg_sub | *Megachile subtranquilla* | Hymenoptera | Molecular ID |
| Meg_umb | *Megachile umbripennis* | Hymenoptera | Molecular ID |
| Meg_azu | *Megascolia azurea* | Hymenoptera | Molecular ID |
| Men_mem | *Menelaides memnon* | Lepidoptera | Morphological ID |
| Men_pol | *Menelaides polytes* | Lepidoptera | Morphological ID |
| Nom_cha | *Nomia chalybeata* | Hymenoptera | Molecular ID |
| Nom_cur | *Nomia curvipes* | Hymenoptera | Molecular ID |
| Nom_inc | *Nomia incerta* | Hymenoptera | Molecular ID |
| Nom_pen | *Nomia penangensis* | Hymenoptera | Morphological ID |
| Nom_wes | *Nomia westwoodi* | Hymenoptera | Molecular ID |
| Pac_ari | *Pachliopta aristolochiae* | Lepidoptera | Morphological ID |
| Pap_bia | *Papilio bianor* | Lepidoptera | Morphological ID |
| Pap_mem | *Papilio memnon* | Lepidoptera | Morphological ID |
| Pap_par | *Papilio paris* | Lepidoptera | Morphological ID |
| Pap_pol | *Papilio polytes* | Lepidoptera | Morphological ID |
| Pap_hel | *Papilio helenus* | Lepidoptera | Morphological ID |
| Par_var | *Parapolybia varia* | Hymenoptera | Molecular ID |
| Par_bad | *Parnara bada* | Lepidoptera | Molecular ID |
| Pel_agn | *Pelopidas agna* | Lepidoptera | Molecular ID |
| Pel_mat | *Pelopidas mathias* | Lepidoptera | Molecular ID |
| Pha_pha | *Phalerimeris phalerata* | Hymenoptera | Morphological ID |
| Phi_loe | *Philanthus loeflingi* | Hymenoptera | Molecular ID |
| Phy_zon | *Phytomia zonata* | Diptera | Molecular ID |
| Pie_rap | *Pieris rapae* | Lepidoptera | Morphological ID |
| Pol_gig | *Polistes gigas* | Hymenoptera | Molecular ID |
| Pol_jap | *Polistes japonicus* | Hymenoptera | Molecular ID |
| Pol_rot | *Polistes rothneyi* | Hymenoptera | Morphological ID |
| Pol_cau | *Polygonia caureum* | Lepidoptera | Morphological ID |
| Pop_mut | *Popillia mutans* | Coleoptera | Morphological ID |
| Pri_bia | *Princeps bianor* | Lepidoptera | Morphological ID |
| Pri_par | *Princeps paris* | Lepidoptera | Morphological ID |
| Rap_ext | *Rapala extensa* | Lepidoptera | Molecular ID |
| Rhy_bru | *Rhynchium brunneum* | Hymenoptera | Molecular ID |
| Sar_kem | *Sarcophaga kempi* | Diptera | Molecular ID |
| Sar_pri | *Sarcophaga princeps* | Diptera | Molecular ID |
| Sar_sco | *Sarcophaga scopariiformis* | Diptera | Molecular ID |
| Sco_bin | *Scolia binotata* | Hymenoptera | Molecular ID |
| Sco_sup | *Scolia superciliaris* | Hymenoptera | Molecular ID |
| Sph_nip | *Sphecodes nipponicus* | Diptera | Molecular ID |
| Sto_dis | *Stomorhina discolor* | Diptera | Molecular ID |
| Sto_obs | *Stomorhina obsoleta* | Diptera | Molecular ID |
| Sua_gre | *Suastus gremius* | Lepidoptera | Molecular ID |
| Syr_ori | *Syritta orientalis* | Diptera | Morphological ID |
| Tel_anc | *Telicota ancilla* | Lepidoptera | Morphological ID |
| Tir_lim | *Tirumala limniace* | Lepidoptera | Morphological ID |
| Tro_aea | *Troides aeacus* | Lepidoptera | Morphological ID |
| Uda_fol | *Udaspes folus* | Lepidoptera | Morphological ID |
| Ves_aff | *Vespa affinis* | Hymenoptera | Morphological ID |
| Ves_bic | *Vespa bicolor* | Hymenoptera | Morphological ID |
| Ves_vel | *Vespa velutina* | Hymenoptera | Morphological ID |
| Ves_fla | *Vespula flaviceps* | Hymenoptera | Molecular ID |
| Xyl_app | *Xylocopa appendiculata* | Hymenoptera | Morphological ID |
| Xyl_nas | *Xylocopa nasalis* | Hymenoptera | Morphological ID |
| Xyl_tra | *Xylocopa tranquebarorum* | Hymenoptera | Morphological ID |
| Ypt_bal | *Ypthima balda* | Lepidoptera | Morphological ID |
| Ziz_oti | *Zizina otis* | Lepidoptera | Morphological ID |
| Zos_jap | *Zosterops japonicus* | Passeriformes | Morphological ID |
